# MYCMI-7: A Small MYC-Binding Compound that Inhibits MYC: MAX Interaction and Tumor Growth in a MYC-Dependent Manner

**DOI:** 10.1158/2767-9764.CRC-21-0019

**Published:** 2022-03-31

**Authors:** Alina Castell, Qinzi Yan, Karin Fawkner, Wesam Bazzar, Fan Zhang, Malin Wickström, Mohammad Alzrigat, Marcela Franco, Cecilia Krona, Donald P. Cameron, Cecilia Dyberg, Thale Kristin Olsen, Vasiliki Verschut, Linnéa Schmidt, Sheryl Y. Lim, Loay Mahmoud, Per Hydbring, Sören Lehmann, Laura Baranello, Sven Nelander, John Inge Johnsen, Lars-Gunnar Larsson

**Affiliations:** 1 Department of Microbiology, Tumor and Cell Biology, Karolinska Institutet, Stockholm, Sweden.; 2 Department of Women's and Children's Health, Karolinska Institutet, Stockholm, Sweden.; 3 Department of Immunology, Genetics and Pathology, The Rudbeck Laboratory, Uppsala University, Uppsala, Sweden.; 4 Department of Cell and Molecular Biology, Karolinska Institutet, Stockholm, Sweden.; 5 Department of Medicine, Karolinska University Hospital, Huddinge, Sweden.

## Abstract

Deregulated expression of *MYC* family oncogenes occurs frequently in human cancer and is often associated with aggressive disease and poor prognosis. While MYC is a highly warranted target, it has been considered “undruggable,” and no specific anti-MYC drugs are available in the clinic. We recently identified molecules named MYCMIs that inhibit the interaction between MYC and its essential partner MAX. Here we show that one of these molecules, MYCMI-7, efficiently and selectively inhibits MYC:MAX and MYCN:MAX interactions in cells, binds directly to recombinant MYC, and reduces MYC-driven transcription. In addition, MYCMI-7 induces degradation of MYC and MYCN proteins. MYCMI-7 potently induces growth arrest/apoptosis in tumor cells in a MYC/MYCN-dependent manner and downregulates the MYC pathway on a global level as determined by RNA sequencing. Sensitivity to MYCMI-7 correlates with MYC expression in a panel of 60 tumor cell lines and MYCMI-7 shows high efficacy toward a collection of patient-derived primary glioblastoma and acute myeloid leukemia (AML) *ex vivo* cultures. Importantly, a variety of normal cells become G_1_ arrested without signs of apoptosis upon MYCMI-7 treatment. Finally, in mouse tumor models of MYC-driven AML, breast cancer, and MYCN-amplified neuroblastoma, treatment with MYCMI-7 downregulates MYC/MYCN, inhibits tumor growth, and prolongs survival through apoptosis with few side effects. In conclusion, MYCMI-7 is a potent and selective MYC inhibitor that is highly relevant for the development into clinically useful drugs for the treatment of MYC-driven cancer.

**Significance:**

Our findings demonstrate that the small-molecule MYCMI-7 binds MYC and inhibits interaction between MYC and MAX, thereby hampering MYC-driven tumor cell growth in culture and *in vivo* while sparing normal cells.

## Introduction

Deregulated expression of the *MYC* family of oncogenes/transcription factors *MYC*, *MYCN,* and *MYCL* (here collectively referred to as “*MYC*”) occurs in more than half of all cancers, and is often strongly associated with aggressive tumors, resistance to therapy, and poor prognosis (1, 2). MYC is a key player in most or all proliferative signaling networks where it acts as a central “hub” for growth signals (3–5), at least partly explaining its role in resistance development to targeted drugs. MYC has therefore been highlighted as a key therapeutic target for cancer therapy for a number of tumor types.

However, currently, there are no specific MYC-targeting drugs available in the clinic. MYC has been considered “undruggable” due to its intrinsically disordered structure and lack of enzymatic activity. MYC functions through interactions with other proteins, such as the heterodimerization partner MAX, which is required for specific binding to “E-box” regulatory elements in the target gene promoters, thereby regulating transcription (3–6). Attempts to find small molecules that interfere with the MYC:MAX or MYC:MAX:DNA interaction, or favor MAX:MAX interaction have been made previously (7–16). Although promising, these attempts have not yet resulted in molecules that have reached the clinic, sometimes due to low potency, poor or unclear target selectivity, and/or inadequate bioactivity *in vivo* (reviewed in refs. 17–21), warranting further efforts to discover and characterize MYC-inhibitory molecules with potential for clinical development. Recently, based on the dominant-negative Omomyc approach, cell-penetrating peptides that bind MYC and blocks MAX binding have been developed (22), and are now in phase I/II clinical trials (NCT04808362). Most likely, it will be beneficial to develop many different strategies to successfully combat this multifunctional target in future cancer treatment. In addition, combinatorial use of anti-MYC drugs with different mechanism of action could potentially synergize and give less side effects by lowering the concentrations of each drug.

Using a cell-based MYC:MAX interaction inhibitor screen, we previously identified several compounds that target the MYC:MAX protein–protein interaction in cells, named MYCMIs. Three of these, MYCMI-6, MYCMI-11, and MYCMI-14, have been characterized previously (8). In the same screen, the compound MYCMI-7 was identified, which in contrast to MYCMI-6, MYCMI-11, and MYCMI-14, also downregulates MYC protein expression and therefore potentially has higher therapeutic efficacy. Here, we characterized the molecule MYCMI-7 in more detail and found that it is a MYC-binding compound that inhibits the MYC:MAX protein interaction in cells, and increases MYC protein turnover. MYCMI-7 induces apoptosis in a MYC-dependent manner in tumor cells but not in normal cells at single-digit micromolar concentrations and exhibits potent tumor growth–inhibitory activity *in vivo* in mouse models of MYC-driven leukemia, breast cancer, and neuroblastoma.

## Materials and Methods

### Cell Culture

The following cell lines were purchased from the ATCC repository during 2014; MCF7 breast carcinoma (ATCC catalog no. HTB-22, RRID: CVCL_0031), MDA-MB-231 breast carcinoma (ATCC catalog no. CRM-HTB-26, RRID: CVCL_0062), and A375 melanoma cells (ATCC catalog no. CRL-1619, RRID: CVCL_0132). All other cell lines were kindly provided by Per Kogner [SK-N-F1 (ATCC catalog no. CRL-2142, RRID:CVCL_1702, SK-N-RA (RRID:CVCL_AQ53), SK-N-AS (ATCC catalog no. CRL-2137, RRID:CVCL_1700), Kelly (DSMZ catalog no. ACC-355, RRID:CVCL_2092), IMR-32 (DSMZ catalog no. ACC-165, RRID:CVCL_0346), and SK-N-DZ (ATCC catalog no. CRL-2149, RRID:CVCL_1701) neuroblastoma cells; 2007], John Sedivy [Rat1 immortalized fibroblasts TGR1 (RRID:CVCL_T955), HO15.19 (RRID:CVCL_0311) and HOMyc3; 2005], Dirk Eick [P493–6 B-cells (RRID:CVCL_6783) 2011], Kenneth Nilsson [Daudi ATCC catalog no. CCL-213, RRID:CVCL_0008, CA46 (ATCC catalog no. CRL-1648, RRID:CVCL_1101), and Mutu (RRID:CVCL_7202) Burkitt's lymphoma, and U-937-GTB (RRID:CVCL_7202) histiocytic lymphoma cells; during 1997], Marie Arsenian Henriksson [primary rat embryonic fibroblasts (REF); 2008], Ludger Hengst [HeLa S3 (ATCC catalog no. CRL-7924, RRID:CVCL_0058) cervix carcinoma; 2002], Bernhard Lüscher [U2OS (ATCC catalog no. HTB-96, RRID:CVCL_0042) osteosarcoma cells, COS-7 simian kidney cells, HEK293 (ATCC catalog no. CRL-1651, RRID:CVCL_0224) human embryonic kidney cells; during 1997], Bert Vogelstein [HCT116 (ATCC catalog no. CCL-247, RRID:CVCL_0291) colon carcinoma and *FBXW7*^−/−^ (RRID:CVCL_HD78) and *p53*^−/−^ (RRID:CVCL_HD97) sublines; 2007], Cristian Nielsen and Damien Hudson (HCT116TOP2A_mAID cells; ref. 23; 2018), and Martin Eilers (U2OS-MYCER osteosarcoma cells; ref. 24; 2004). HeLa, MCF7, MDA-MB-231, U2OS, HCT116 (and sublines), SK-N-F1, SK-N-RA, SK-N-AS, A375, COS-7, Rat1 fibroblasts (TGR1, HO15.19, and HOMyc3), primary REFs, and HEK293 cells were maintained in DMEM supplemented with 10% FBS and 1% penicillin/streptomycin. Kelly, IMR-32, SK-N-DZ, P493-6, Daudi, CA46, Mutu, and U-937 were kept in RPMI supplemented with 10% FBS and 1% penicillin/streptomycin. In addition, the medium for HO15.19 and primary REFs contained 1% sodium pyruvate. U2OS-MYCER cell lines were cultured in phenol-red free DMEM supplemented with 10% estrogen-free FBS and 1% penicillin/streptomycin and treated with 100 nmol/L 4-hydroxytamoxifen (4-OHT; Sigma-Aldrich) to activate MYCER. HEK293 cell lines were established to stably express both full-length MYC and MAX fused to split Guassia luciferase fragments GLuc1 and GLuc2, respectively. To induce TOP2 degradation in HCT116TOP2A_mAID cells, the culture medium was replaced with DMEM containing 500 μmol/L freshly prepared auxin (Indole-3-acetic acid, Sigma). All cells used were *Mycoplasma* free and kept at 37°C and 5% CO_2_. Cells were used for a maximum of 10 passages after collection or thawing. All cell lines were periodically checked for *Mycoplasma* contamination using MycoAlert Mycoplasma Detection Kit (Lonza, catalog no.: LT07–318) following the manufacturer's instructions. The human neuroblastoma cell lines were authenticated by short tandem repeat (STR) analysis (Eurofins Genomics).

Cell growth and viability was estimated in triplicates with WST-1 (Roche) or Resazurin sodium salt (Sigma-Aldrich) assays in medium at 37°C and 5% CO_2_ for 2 hours after which absorbance or fluorescence, respectively, was measured with an Omega Fluostar (BMG Labtech) in a 96-well plate format. For further details, see Supplementary Information.

### Patient-Derived Cells

Tumor sample collection underlying patient-derived glioblastoma and leukemia cell cultures were approved by the Uppsala regional ethical review board (numbers 2007/353, 201905861) and the ethical review board in Stockholm (2010/1893–31), respectively. Written informed consent was obtained from all subjects included. The studies were performed according to the Declaration of Helsinki.

### Compounds

MYCMI-7 (2-(5,11-Dimethyl-6H-25-pyrido[4,3-b]carbazol-2-yl)-N,N-diethy-lethanamine acetate, NSC-359449) was synthesized by Honghui-Meditech, purity was >95% determined by high-performance liquid chromatography. DMSO, JQ1, 10058-F4, ellipticine, doxorubicin, etoposide, and camptothecin were purchased from Sigma-Aldrich. All compounds were dissolved in DMSO (Sigma-Aldrich) to a final concentration of 10 mmol/L, verified by mass spectrometry (LC-MS) and stored in −80°C for further use.

### *Gaussia* Luciferase Protein Fragment Complementation Assay

The Gaussia luciferase protein fragment complementation assay (GLuc) has been described elsewhere (8, 25). Each GLuc construct (0.2–0.4 μg) were used together with 0.05 μg pCMV-LUC (firefly luciferase) per transfection in 12-well plates. Twenty-four hours later, cells were treated with compound or DMSO. After another 17 hours, the cells were harvested, and luciferase activity measured using the Dual Luciferase Kit (Promega) in a Berthold Lumat LB9501 or OmegaFluostar (BMG Labtech). For further details, see Supplementary Data.

### *In Situ* Proximity Ligation Assay

The *in situ* proximity ligation assay (isPLA) has been described previously (8). Briefly, cells were grown on collagen-coated chamber slides (Falcon), treated with compounds, and then washed twice with PBS and fixed in ice-cold methanol for 5–15 minutes at room temperature. Slides were washed in PBS with 0.05% Tween 20 and incubated in blocking buffer after which isPLA was performed using the Duolink *In Situ* PLA Kit (Sigma-Aldrich) according to the manufacturer's protocol. DNA was stained with DAPI. Incubation with primary antibodies were performed at +4**°**C overnight. Images were taken using an Axiovert 200M inverted microscope (Zeiss) and fluorescent dots were quantified using semiautomated analysis in ImageJ (http://imagej.net) and averaged to the number of dots per cell. Antibodies used are listed in Supplementary Data.

### Western Blot, Coimmunoprecipitation, Chromatin Immunoprecipitation, and Cycloheximide Chase

Western blot, immunoprecipitations, and cycloheximide chase were performed essentially as described in ref. 26. For Western blot analysis, equal amounts of proteins (30–50 μg) were separated on a 4%–12% SDS-PAGE gel. Proteins were transferred to Immobilion-P membrane (Millipore) and detected by immunostaining and chemiluminescence (Immobilion Western HRP Substrate; Millipore). For immunoprecipitation, 500–1000 μg of protein was used. Chromatin immunoprecipitation was performed as described previously (27). Briefly, cells were crosslinked with 1% formaldehyde on ice for 6 minutes. Nuclear chromatin was sonicated on ice to fragments from 0.3 kb to 0.5 kb. Nuclear chromatin equivalent to 2.5 × 10^7^ cells was immunoprecipitated with 2 µg antibody. For studies of protein turnover, cells were treated with 100 µg/mL cycloheximide to block protein synthesis for 2 hours, followed by chase. Quantification of western blots was done by Image J analysis.

### Surface Plasmon Resonance

The surface plasmon resonance (SPR) experiments were performed at 25°C using a Biacore T200 (GE Healthcare) instrument kindly provided by SciLifeLab Solna. An amino coupling procedure was used to immobilize protein on a CM5 sensor chip (GE Healthcare). Sensorgrams were generated by subtraction of the reference (blank immobilized) surface. For further details, see Supplementary Information.

### RNA-Sequencing Analysis

Libraries for RNA sequencing were prepared using the TruSeq Stranded Total RNA kit with RiboZero (Illumina) and sequenced on two lanes of the HiSeq 2500 platform with a single-end 1 × 51 setup and the HiSeq Rapid SBS v2 chemistry. Demultiplexed .fastq files were aligned to the human GRCh37 reference genome using Tophat v 2.0. After alignment, .bam files from two separate flowcell lanes were merged using samtools. Raw read counts per gene were then generated using htseq-count v0.6.1. Differential expression analysis comparing the two DMSO-treated to the two MYCMI-7–treated samples was performed using the R/Bioconductor DESeq2 package v1.26.0 (Bioconductor v3.10, R v3.6.1), Following differential expression analysis, all genes were ranked according to *P*_adj_ value and log fold change. Gene-set enrichment analysis was then performed using GSEA software with the Hallmarks (H) and curated (C2:CGP) MSigDB gene sets; v6.2.

### Mouse Tumor Models

All animal protocols in these studies were approved by the ethical committee for animal experiments of northern Stockholm (N47/14, N241/15 and N231/14) and of Uppsala (C41/14). Mice were maintained under pathogen-free conditions according to guidelines of the animal facility at MTC, Karolinska Institutet, or at AKM, Karolinska University Hospital. Drug toxicity in the mice was evaluated by examining changes in body weight, changes in behavior, and overall wellness, loss of fur coat, breathing, mouse activity, and body posture following drug injections, food intake, as well as the histologic effect on the livers of treated mice.

For the AML tumor model, MYC+BCL-XL expressing, GFP^+^ leukemic cells were isolated from leukemic mice as described previously (28). A total of 3 × 10^5^ cells were injected into each recipient C57BL mouse after irradiation (600 rad). AML-like leukemia initiation was confirmed via flow cytometry and Giemsa staining, at day 8 after transplantation. MYCMI-7 cells were then administrated intraperitoneally daily at a dose of 12.5 mg/kg. Liver, spleen, and bone marrow were extracted at day 11, 15, and endpoint for measurement. In addition, body weight of mouse in each group was also collected every fourth day until the endpoint of the experiment.

For the xenograft transplantation mouse tumor models of breast cancer and neuroblastoma, 6–8 weeks old NOD/SCID mice (Taconic) or NMRI nu/nu (Scanbur) were injected subcutaneously with 5 × 10^6^ MDA-MB-231 breast cancer cells or *MYCN*-amplified SK-N-DZ neuroblastoma cells. When tumors reached a size of 100–200 mm^3^, mice started receiving treatment (6.25 mg/kg MYCMI-7) administered intratumorally (50 μL) twice per week. The last dose was administered 3 hours before termination. Mice were sacrificed when the tumors had reached a size of 1,000 mm^3^ (MDA-MB-231) or 1,500 mm^3^ (SK-N-DZ). Tumors were collected and frozen in optimal cutting temperature (Cryomount, Histolab) or fixed in buffered 4% formaldehyde solution (Histolab) and paraffin-embedded.

For description of IHC and immunofluorescence, see Supplementary Data.

### Statistical Analysis

Analysis of the probability of a cell line with “high MYC” or “low MYC” mRNA/protein levels to respond to MYCMI-7 among the NCI-60 cancer cell lines was tested with the binomial exact test. The analysis was carried out in R (v. 3.3.3; R Foundation for Statistical Computing), at a level of significance α = 0.05. The Kaplan-Meier survival curves in the animal studies were evaluated with log-rank test using GraphPad Prism. The rest of the data were analyzed with two-tailed paired Student *t* tests (GraphPad Prism).

### Data Availability Statement

The RNA-seq data in this study are publicly available in Gene Expression Omnibus (GEO) at GSE197062. The other data generated in this study are available within the article and its Supplementary Data files or upon request from the corresponding author.

## Results

### MYCMI-7 Inhibits the MYC:MAX Interaction in Cells, Binds MYC *In Vitro*, and Blocks MYC Function

**FIGURE 1:**
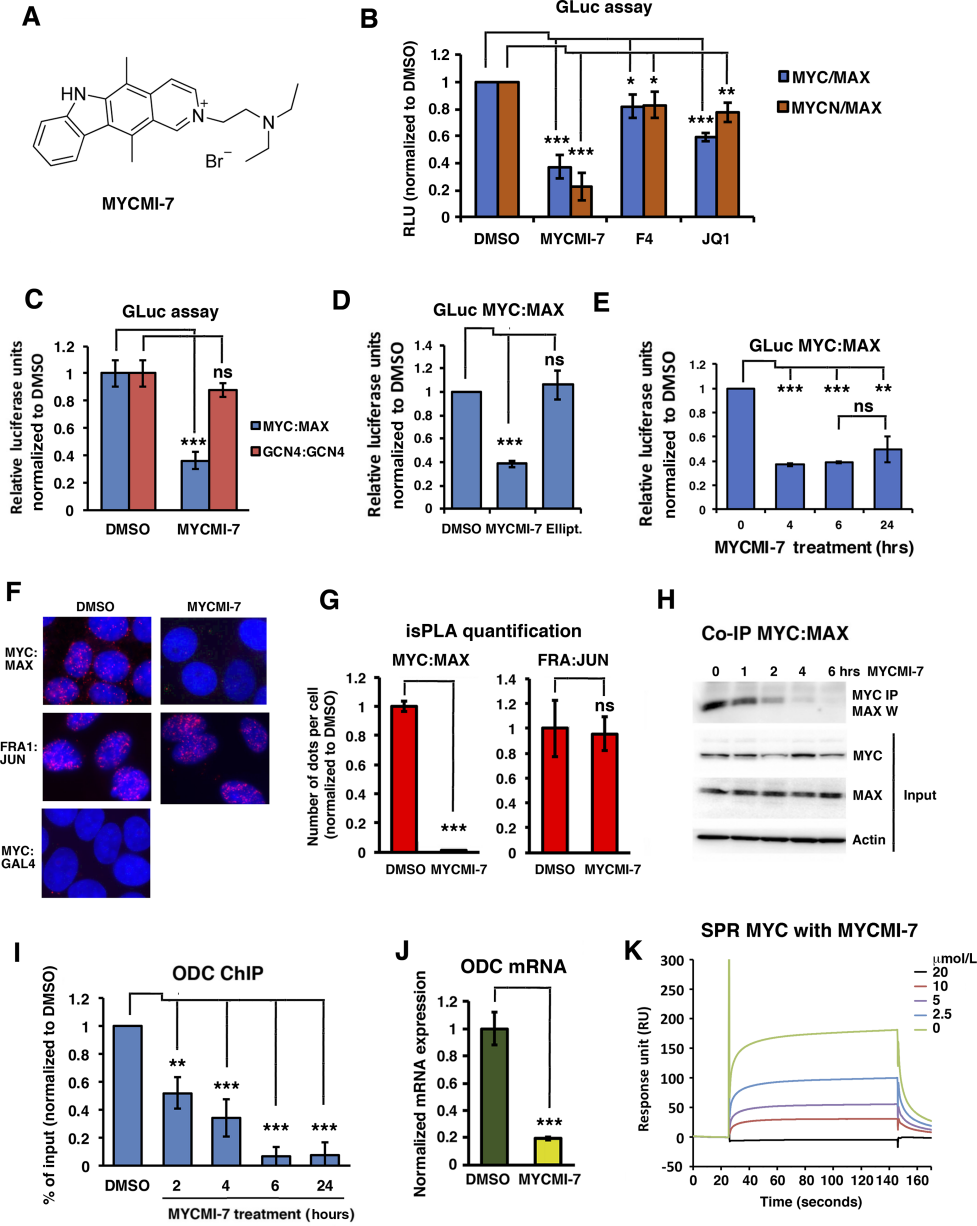
MYCMI-7 inhibits the MYC:MAX interaction in cells, binds MYC *in vitro*, and blocks MYC function. **A,** Molecular structure of MYCMI-7. **B**–**E,** Split Gaussia luciferase (GLuc) assay. In **B**–**D,***MYC* or *MYCN* and *MAX* GLuc constructs were cotransfected into HeLa cells together with a firefly luciferase vector. Twenty-four hours posttransfection, cells were treated with compounds, and after another 17–24 hours cells were harvested and both GLuc and firefly luciferase activities were measured. The mean of at least three different experiments is shown. Treatment of transfected cells with: 5 μmol/L of MYCMI-7, 64 μmol/L 10058-F4 or 5 μmol/L JQ1, respectively, as indicated (**B**). *P* values for DMSO versus MYCMI-7, 10058-F4, or JQ1 for MYC:MAX; 0.00024, 0.0205, and 2.50 × 10^−5^, respectively, and for MYCN:MAX; 4.83 × 10^−9^, 0.020, and 0.0022, respectively. **C,** Treatment of cells expressing MYC:MAX GLuc or GCN4:GCN4 GLuc with 6.5 μmol/L MYCMI-7. *P* values for DMSO versus MYCMI-7 for MYC:MAX and GCN4:GCN4; 0.0010 and 0.0539, respectively. **D,** Treatment of cells expressing MYC:MAX with 5 μmol/L MYCMI-7 or 5 μmol/L ellipticine. *P* values DMSO versus MYCMI-7 or ellipticine; 1.68 × 10–6 and 0,332, respectively. **E,** A HEK293 cell clone stably coexpressing MYC-Luc1 and MAX-Luc2 exposed to 5 μmol/L MYCMI-7 for indicated time points, after which cells were harvested and GLuc assay was performed. *P* values DMSO versus MYCMI-7 at 4, 6, and 24 hours; 2.0 × 10^−7^, 5.3 × 10^−8^, 0.0012, respectively. *P* value MYCMI-7 during 6 hours versus 24 hours; 0.17. **F,** isPLA. MCF7 breast cancer cells were treated with 5 μmol/L MYCMI-7 for 24 hours, thereafter fixed and subjected to the isPLA assay to detect endogenous MYC:MAX or FRA1:JUN interactions. MYC:GAL4 was used as negative control in the isPLA assay. **G,** Quantification of isPLA dots. *P* values for DMSO versus MYCMI-7 for MYC:MAX or FRA1:JUN; 1.22 × 10^−6^ and 0.888, respectively. **H,** Co-IP of endogenous MYC and MAX proteins (top) from MCF7 cells after treatment with 5 μmol/L MYCMI-7 at various time points. IP, immunoprecipitation; W, Western blot. The MYC, MAX, and actin input are shown in panels 2–4 from the top. **I,** ChIP of MYC at the *ODC1* target gene promoter after treatment of MCF7 cells with 5 μmol/L MYCMI-7 for indicated time points. *P* values for DMSO versus MYCMI-7 at 2, 4, 6, and 24 hours; 0.0011, 0.00010, 1.82 × 10^−7^ and 8.23 × 10^−5^, respectively. **J,** MYC target gene *ODC1* mRNA expression levels in MCF7 cells 24 hours after treatment with 5 μmol/L MYCMI-7, quantified by qPCR. *P* value for DMSO versus MYCMI-7; 0.00029. The statistical analysis in **B**–**J** were performed by *t* test. **K,** Recombinant MYC bHLHZip protein domain immobilized onto a CM5 chip was subjected to various concentrations of MYCMI-7 and the complex of MYCbHLHZip-MYCMI-7 was measured as response units. The sensogram was produced by subtracting the blank response. *, *P* < 0.05; **, *P* < 0.01; ***, *P* < 0.001; ns, not significant.

We recently identified MYCMI-7 (2-(5,11-Dimethyl-6H-25-pyrido[4,3-b]carbazol-2-yl)-N,N-diethylethanamine acetate, NSC-359449; Fig. 1A) in a cell-based MYC:MAX interaction inhibitor screen in HEK293 cells (8). To verify that MYCMI-7 inhibits MYC:MAX dimerization in tumor cells, we utilized the split *Gaussia princeps* luciferase (GLuc) assay, with GLuc fragments GLuc1 and GLuc2 fused to MYC (or MYCN) and MAX, respectively (8, 25), in HeLa cells. MYCMI-7 (5 μmol/L) treatment inhibited both the MYC:MAX and MYCN:MAX Gluc interactions significantly, whereas the previously published MYC:MAX inhibitor 10058-F4 (64 μmol/L; ref. 16) and the bromodomain inhibitor JQ1 (5 μmol/L; ref. 29) had weaker, but also significant, effects (Fig. 1B). In contrast, MYCMI-7 did not have any significant effect on homodimerization of the bZip protein GCN4 (Fig. 1C). Although MYCMI-7 shows structural similarities to ellipticine, the latter was inactive at the same concentration (Fig. 1D; Supplementary Fig. S1A) and was hereafter used as a reference compound. The effect of MYCMI-7 was rapid; when the MYC:MAX interaction was measured by GLuc assay in HEK293 cells stably coexpressing MYC-GLuc1 and MAX-GLuc2, it was inhibited already at the first time point (4 hours) of treatment and thereafter (Fig. 1E). To validate the effect of MYCMI-7 on endogenous MYC:MAX interactions, isPLA (8, 30) was used in MCF7, a breast cancer cell line we have used extensively to validate the effect of MYC:MAX inhibitors on endogenous MYC:MAX interactions (8). Treatment with 5 μmol/L MYCMI-7 strongly inhibited the MYC:MAX interaction in these cells, but not the interaction between the bZip proteins FRA1 and JUN (Fig. 1F and G). In addition, co-immunoprecipitation (co-IP) performed in the same cells showed that the MYC:MAX interaction was significantly decreased already at 1 hour after treatment, and was reduced drastically thereafter with a minimum at 6 hours, while having much less effect on the total level of MYC and MAX at these time points (Fig. 1H; Supplementary Fig. S1B–D). Furthermore, chromatin immunoprecipitation (ChIP) in MCF7 cells showed that association of MYC with the *ODC1* gene promoter, a well-known MYC target gene, was reduced by MYCMI-7 treatment (5 μmol/L), starting already at the first time point (2 hours) and reaching a minimal level from 6 hours onwards (Fig. 1I), consistent with the reduced *ODC1* expression in these cells after 24 hours of treatment (Fig. 1J). Binding of MYC to the *ODC1* promoter was also reduced in U-937 myeloid cells after MYCMI-7 treatment (Supplementary Fig. S1E).

To further investigate potential direct binding of MYCMI-7 to MYC, an equilibrium SPR assay was carried out using the recombinant bHLHZip domain of the MYC protein. A dose-dependent increase in MYCMI-7 binding was observed, with association and dissociation curves indicating that MYCMI-7 binds directly to MYC with a *K*_d_ of approximately 4 μmol/L as determined by Langmuir 1:1 analysis (Fig. 1K).

We concluded that MYCMI-7 binds MYC *in vitro*, and that it rapidly, strongly, and selectively inhibits MYC:MAX interaction and MYC's association with chromatin in cells at low micromolar concentrations.

### MYCMI-7 Increases MYC Protein Turnover

**FIGURE 2:**
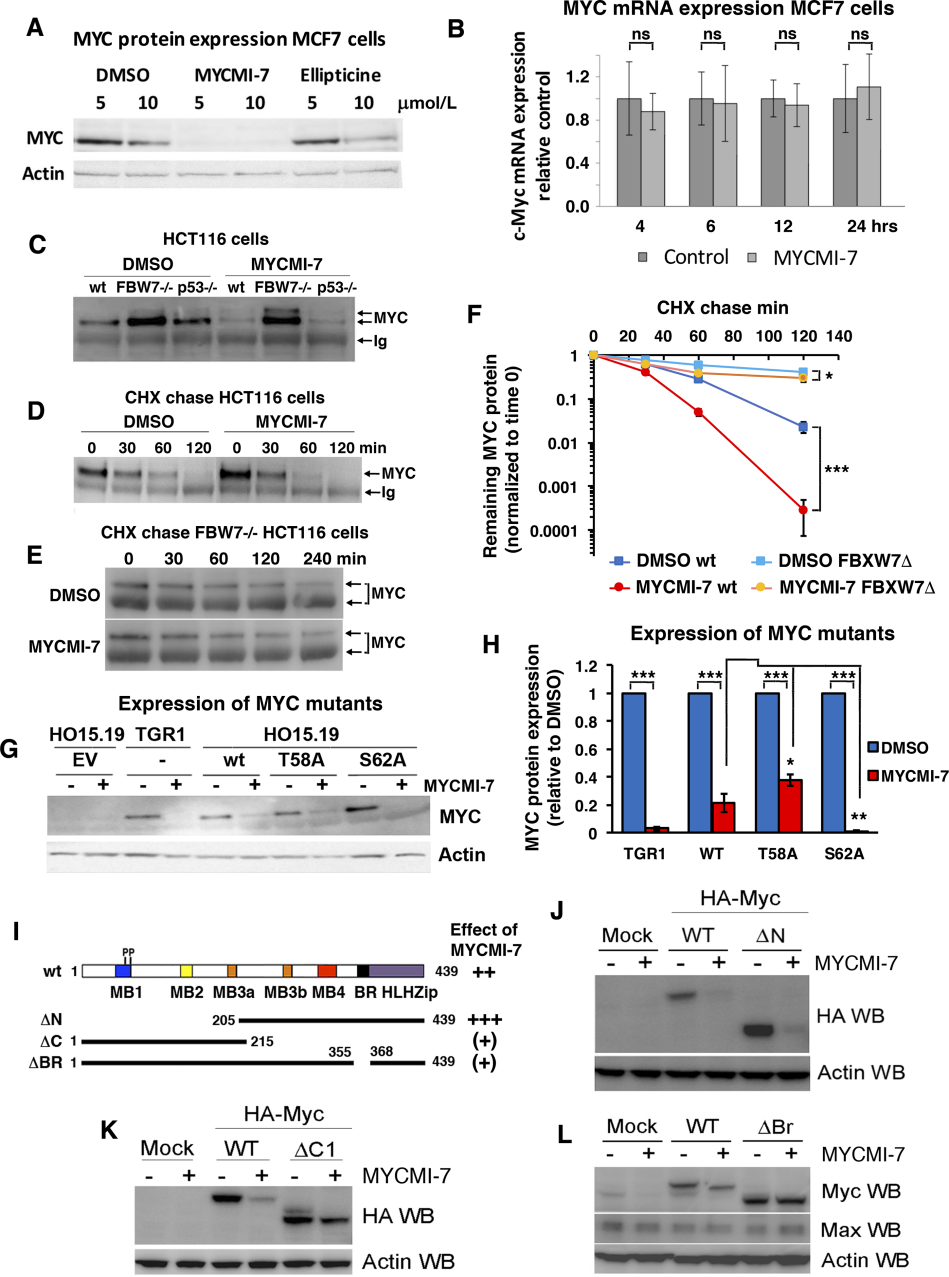
MYCMI-7 downregulates MYC and MYCN protein levels. **A,** Steady-state levels of MYC protein in MCF7 cells after 17 hours exposure to indicated concentrations MYCMI-7 or ellipticine. **B,***MYC* mRNA expression in MCF7 cells after treatment with MYCMI-7 for various time points. **C,** Steady-state levels of MYC protein in different HCT116 colon carcinoma cell lines (WT, *FBXW7*^−/−^ or *p53*^−/−^) after 17-hour exposure to 5 μmol/L MYCMI-7. Endogenous MYC was immunoprecipitated and detected by Western blot analysis. **D,** Cycloheximide (CHX) chase in HCT116 cells. Cells were subjected to 5 μmol/L MYCMI-7 30 minutes prior cycloheximide addition. Cells were harvested at the indicated time points, after which MYC was immunoprecipitated, and the MYC levels analyzed using Western blot. MYC was immunoprecipitated in **C** and **D** to ensure detection of low levels of MYC after MYCMI-7 treatment and after chase to enable quantification of differences in MYC turnover rate. **E,** Cycloheximide chase after MYCMI-7 treatment performed as in **D** using *FBXW7*-deficient HCT116 cells. The upper MYC band more readily observed after *FBXW7* loss (**C** and **E**) likely represent a MYC subpopulation that is stabilized under these conditions. **F,** Quantification of cycloheximide chase experiments shown in **D** and **E**. Note the logarithmic scale on the *y*-axis. *P* values for DMSO versus MYCMI-7 for HCT116 wt and *FBXW7*^−/−^ cells at cycloheximide chase for 120 hours; 0.00086 and 0.024, respectively. **G,** Stable expression of wt, T58A or S62A MYC in Rat1 HO15.19 *MYC*^−/−^ cells. The cells were treated with 5 μmol/L MYCMI-7 overnight, and MYC expression was analyzed by Western blot. Rat1 TGR1 cells (parental to HO15.19) was used as reference. **H,** Quantification of MYC expression shown in **G** normalized to actin expression. *P* values for DMSO versus MYCMI-7 for TGR1, HO15.19 wt, T58A, and S62A cells; 3.97 × 10^−8^, 4.79 × 10^−5^, 1.34 × 10^−5^, and 6.00 × 10^−9^, respectively. *P* values for MYCMI-7 treatment of HO15.19 wt versus T58A or S62A cells; 0.027 and 0.0089, respectively. The statistical analysis in **F** and **H** were performed by *t* test. **I,** Cartoon showing a linear structural representation of wt MYC and indicated mutants. **J**–**L,** Expression of indicated MYC mutants after MYCMI-7 treatment. COS-7 cells were transfected with CMV-driven vectors containing HA-tagged wt *MYC* or mutants for 24 hours and treated with 5 μmol/L MYCMI-7 for 17 hours. The ΔN (**J**) and ΔC (**K**) *MYC* mutants encodes the C-terminal and N-terminal halves of the MYC protein, respectively. ΔBr (**L**) lacks the DNA-binding basic region of the protein (**A**–**L**). Representative Western blots out of at least three experiments are shown. *, *P* < 0.05; **, *P* < 0.01; ***, *P* < 0.001; ns, not significant.

We next studied the expression levels of MYC and MAX after MYCMI-7 treatment. The steady-state level of the MYC protein decreased drastically in MCF7 cells after 17-hour treatment with 5 μmol/L MYCMI-7, while treatment with 5 μmol/L ellipticine had no effect (Fig. 2A). Considering the structural similarities between the two compounds, we increased the dose to 10 μmol/L, but ellipticine still only had a slight effect on MYC protein level. Also, DMSO alone had a some effect on MYC expression at 10 μmol/L, although this is not something we observe generally. Downregulation of MYC or MYCN protein expression upon MYCMI-7 treatment was also confirmed in HeLa, P493-6, HCT116 cells, and in MYCN-amplified Kelly neuroblastoma cells (Supplementary Fig. S2A–D). P493-6 is a B-cell line harboring a doxycycline-regulated MYC transgene (31), and here treatment with 5 μmol/L MYCMI-7 for 17 hours reduced MYC expression to the same extent as turning off MYC expression by addition of doxycycline (Supplementary Fig. S2D). In contrast, the expression of MAX was not affected in these cell lines, except in Kelly neuroblastoma cells where MAX protein level was reduced following treatment with MYCMI-7. Reduction of MAX was observed in some additional neuroblastoma cell lines, apparently irrespective of MYCN amplification status (Supplementary Fig. S2E). The decrease in MYC protein level was not due to reduced *MYC* mRNA expression as determined by qRT-PCR in MCF7 cells (Fig. 2B). We therefore looked at MYC protein stability using HCT116 cells with knockout of *FBXW7*, encoding the main E3 ubiquitin ligase targeting MYC (32, 33). As excepted, these cells displayed an increased MYC protein level (Fig. 2C; Supplementary Fig. 2F). Interestingly, the MYC protein level was strongly reduced in wt HCT116 cells as well as in *p53*-null HCT116 cells (used as a reference), but much less affected in the *FBXW7*^−/−^ cells after MYCMI-7 exposure (Fig. 2C). Furthermore, cycloheximide (CHX) chase assay showed that the half-life of the MYC protein decreased significantly in wt HCT116 cells in response to MYCMI-7 from approximately 19 minutes to 8.5 minutes (Fig. 2D and F). There was a significant increase in MYC turnover rate also in *FBXW7*^−/−^ HCT116 cells, but the relative difference in half-life was smaller; from approximately 93 minutes to 73 minutes (Fig. 2E and F). This suggests that MYCMI-7 speeds up FBXW7-dependent degradation of MYC, but additionally indicates that other factors are also involved. We next investigated whether phosphorylation of MYC at Thr-58 or Ser-62, which enable binding of FBXW7 to MYC and regulate FBXW7-mediated MYC turnover, change in response to MYCMI-7. However, there was no apparent difference in the ratio between phosphorylated versus total MYC in wt or *FBXW7*^−/−^ HCT116 cells after MYCMI-7 treatment (Supplementary Fig. S2F and S2G). To further investigate the role of the Thr-58 and Ser-62 phosphorylation sites, HO15.19 *MYC* knockout Rat1 cells were reconstituted with T58A or S62A MYC mutants, or wt MYC as control. MYCMI-7 treatment drastically reduced the levels of both wt and the MYC mutants (Fig. 2G and H), but the reduction in MYC level was significantly lower for the T58A MYC mutant and significantly higher for the S62A MYC mutant. Similar results were obtained in U2OS cells transiently transfected with vectors expressing wt *MYC* and T58A or S62A *MYC* mutants, although the effects were less pronounced, possibly due to overexpression (Supplementary Fig. S2H). This suggests that MYC phosphorylation status affects MYCMI-7–mediated degradation of MYC to a certain degree, but that MYCMI-7 also affects MYC stability through other mechanisms. Furthermore, MYCMI-7 reduced MYC levels even further in Rat1 TGR1 cells (parental cells to the HO15.19 *MYC* knockout cells), suggesting that regulatory elements in the 5′ or 3′ ends of the *MYC* transcript, lacking in the *MYC* transgenes, may play a posttranscriptional role in regulation of MYC protein levels after treatment. This remains to be investigated in the future.

To map regions of MYC involved in MYCMI-7–mediated MYC turnover, different *MYC* mutants were utilized in transient transfection assays (Fig. 2I). This analysis showed that the C-terminus, but not the N-terminus, of MYC was required for turnover. Further mapping of the C-terminal part showed that the DNA-binding basic region was necessary for MYCMI-7–mediated MYC turnover (Fig. 2J–L).

### MYCMI-7 Reduces Tumor Cell Growth and Viability in a MYC-Dependent Manner and Downregulates the MYC Pathway

**FIGURE 3:**
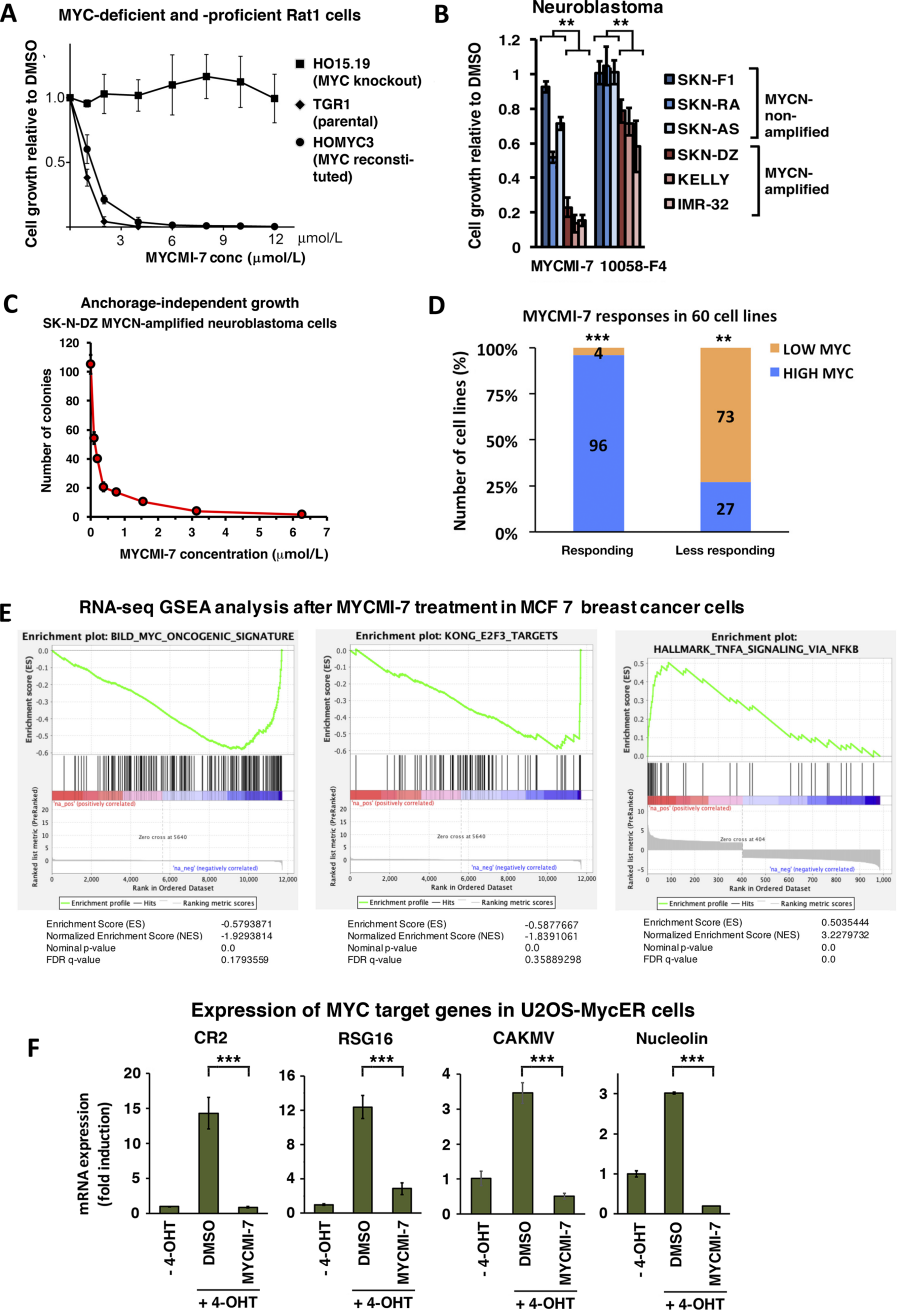
MYCMI-7 reduces tumor cell growth/viability in a MYC-dependent manner and downregulates the MYC pathway. **A,** Cell growth of immortalized Rat1 fibroblasts with different MYC status; TGR1 (wt *MYC*), HO15.19 (*MYC*^−/−^) and HOMyc3 (HO15.19 with reconstituted MYC) after treatment with the indicated concentrations of MYCMI-7 for 48 hours. **B,** Cell growth of human neuroblastoma cells with or without *MYCN*-amplification after treatment with 6.25 μmol/L MYCMI-7 or 64 μmol/L 10058-F4 for 24 hours. *P* values for *MYCN*-amplified versus *MYCN*-nonamplified cell lines MYCMI-7 and 10058-F4; 0.010 and 0.0059, respectively, by *t* test. **A** and **B,** Cell growth/viability was measured using the WST-1 assay. An average of three different experiments performed in triplicates is shown. **C,** 3D cultures of SKN-DZ neuroblastoma cells with *MYCN* amplification exposed to indicated concentrations of MYCMI-7 for 2 weeks. Colony formation in agarose was detected by MTT staining and quantified. **D,** Correlation of MYCMI-7 response (GI_50_) with *MYC* mRNA levels of the NCI-60 human tumor cell lines extracted from CellMiner and complemented with MYC protein levels from the Novartis proteome scout project or from the literature (8). “Responsive” and “less responsive”; cell lines with positive and negative log_10_ GI_50_ values, respectively. “Higher MYC” and “lower MYC”; cell lines with higher and lower MYC expression levels (MYC mRNA/protein) than average, respectively. *P* values “high” versus “low” expression for “responsive” and “less responsive” cell lines; 1.55 × 10^−6^ and 0.007, respectively, assessed using binomial test. **E,** Gene-set enrichment analysis (GSEA) of RNA-seq data from MCF7 breast carcinoma cells treated with 5 μmol/L MYCMI-7 for 24 hours. **F,** Inhibition of MYC transactivation of target genes *CR2*, *RSG16*, *CAKMV*, and *nucleolin* as determined by qRT-PCR analysis, based on three biological experiments with three technical repeats each. U2OS-MYCER cells were treated with (MYC ON) or without (MYC OFF) 100 nmol/L 4-OHT for 4 hours, after which DMSO or MYCMI-7 (5 μmol/L) were added for 24 hours before total RNA was extraction. Fold changes in mRNA expression are presented relative to DMSO in MYC OFF cells after normalization to *GAPDH*, used as reference gene. *P* values DMSO versus MYCMI-7 for *CR2*, *RSG16*, *CAKMV* and *nucleolin*; 4.41 × 10^−7^, 0.0004, 7.41 × 10^−5^, and 7.17 × 10^−9^, respectively, assessed using *t* test. *, *P* < 0.05; **, *P* < 0.01; ***, *P* < 0.001; ns, not significant.

To address whether MYCMI-7 affects cell growth in a MYC-dependent manner, we utilized the immortalized Rat1 fibroblasts with different MYC status mentioned above; the HO15.19 *MYC*-null cells derived from parental TGR1 cells, and HOMyc3, which are HO15.19 cells reconstituted with *MYC* (34). Proliferation/viability of cells expressing MYC declined drastically at low concentrations of MYCMI-7, with an average growth inhibition of 50% (GI_50_) around 2 μmol/L as measured by WST-1 assay (which measures metabolic activity in cells), while the *MYC*-null cells were unaffected even at concentrations of 10 μmol/L (Fig. 3A), demonstrating that the effect of MYCMI-7 was MYC dependent. In contrast, ellipticine reduced growth of all three cell clones in a dose-dependent manner, irrespective of MYC status (Supplementary Fig. S3A). When HO15.19 cells were exposed to MYCMI-7 concentrations higher than 10 μmol/L, a marked decline in viability was observed, suggesting that off-target effects started to appear above this concentration (Supplementary Fig. S3B).

To investigate whether MYCMI-7 affects growth of typical MYC-driven tumor cells, we utilized a panel of childhood neuroblastoma cells with or without *MYCN*-amplification as well as a set of Burkitt's lymphoma cell lines, which carry MYC translocations. Treatment with MYCMI-7 reduced tumor growth and viability in all the neuroblastoma cell lines, but the effect was significantly stronger in the *MYCN*-amplified cases (Fig. 3B). Note that non-*MYCN*–amplified neuroblastoma cells express MYC, albeit at a lower level than MYCN in amplified lines (8). The efficiency of MYCMI-7 toward MYCN-amplified neuroblastoma cells was even stronger in three-dimensional (3D) cultures, with GI_50_ in the nanomolar range (Fig. 3C; Supplementary Fig. S3C). MYCMI-7 also strongly reduced growth of the three Burkitt's lymphoma cell lines (Supplementary Fig. S3D).

To investigate whether the levels of MYC expression in tumor cells correlate with growth-inhibitory response to MYCMI-7, we utilized GI_50_ and mRNA expression data from the NCI-60 diverse human tumor cell line panel available for MYCMI-7 by the Developmental Therapeutics Program of the National Cancer Institute (DTP-NCI). The data was extracted from the NIH-supported CellMiner version 2.1 (https://discover.nci.nih.gov/cellminer) and combined with MYC protein data obtained from Novartis proteome scout SymAtlas Project (https://proteomescout.wustl.edu/proteins/52581/expression) or elsewhere in the literature as described previously (8). The cell lines were categorized as “responsive” or “less responsive” to MYCMI-7 based on average logarithmic GI_50_ values, as well as the categories “higher MYC” and “lower MYC” based on higher or lower than average MYC mRNA and/or high protein levels. There was a significant correlation between the response to MYCMI-7 and the MYC mRNA/protein levels among the 60 tumor cell lines (Fig. 3D). Ninety-six percent of the cells with high MYC mRNA/protein level were responsive to MYCMI-7 and 73% of the cells with low MYC levels were less responsive (Fig. 3D). This indicates that cells with high MYC levels are more likely to respond to MYCMI-7 treatment than cells with low MYC levels.

To investigate whether MYCMI-7 could inhibit MYC-induced oncogenic transformation of normal cells together with H-RAS, normal REFs were transfected with *MYC* + *RAS* vectors. Formation of transformed foci as well as the ability of MYC + RAS–transformed REFs to form colonies in semi-solid medium was strongly inhibited by treatment with MYCMI-7 (Supplementary Fig. S3E and S3F).

To study the impact of MYCMI-7 on global gene expression, we performed RNA-seq analysis in MCF7 breast carcinoma cells after treatment with 5 μmol/L MYCMI-7 for 24 hours. Gene-set enrichment analysis (GSEA) of differentially expressed genes showed a downregulation of the MYC and E2F target genes (Fig. 3E, left and middle). In contrast, upregulated genes were enriched in pathways associated with inflammatory signaling via NFκB, which is consistent with the reported suppressive action of MYC on immune signaling (Fig. 3E, right; refs. 35, 36). To further document the impact of MYCMI-7 on MYC's transcriptional activity, we utilized U2OS cells expressing a MYC-estrogen receptor (MYCER) fusion protein, which is regulated by 4-OHT (24). Treatment with MYCMI-7 significantly reduced 4-OHT–induced expression of *CR2*, *RGS16*, *CAMKV*, and *nucleolin* (Fig. 3F), which all previously have been characterized as direct MYC target genes (37, 38).

Taken together, these results suggest that MYCMI-7 reduces tumor cell growth/viability in a MYC-dependent manner and downregulates the MYC pathway.

### MYCMI-7 Induces Growth Arrest and Apoptosis in Malignant Cells and only Growth Arrest in Normal Cells

**FIGURE 4:**
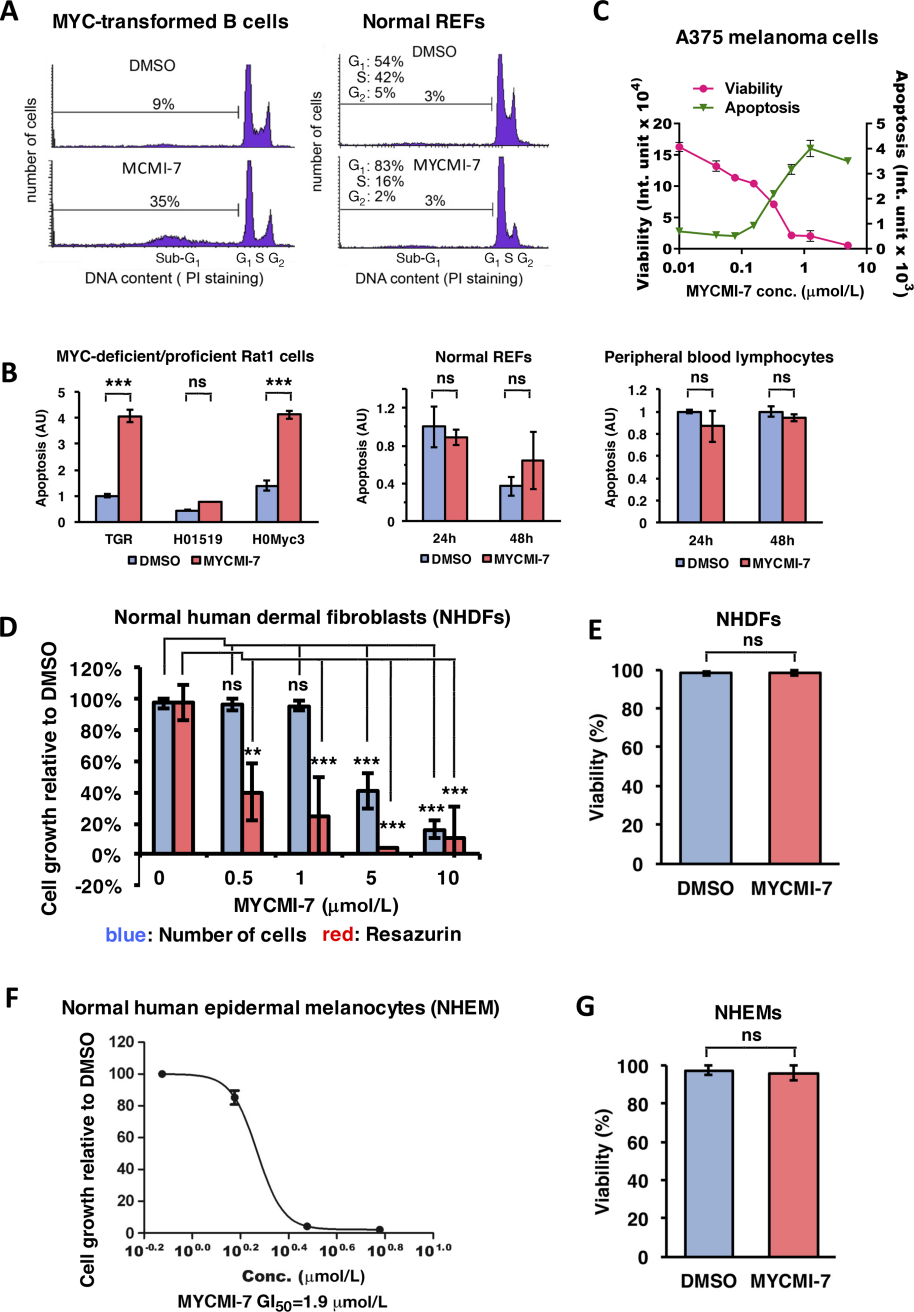
MYCMI-7 induces apoptosis in malignant cells and growth arrest in normal cells. **A,** Cell-cycle distribution analysis by flow cytometry of MYC-regulated P493-6 B cells and normal REFs. Left, P493-6 synchronized in G0 by 24 hours treatment of 1 μg/mL doxycycline. Doxycycline was removed and the cells were harvested 48 hours after the addition of fresh medium containing DMSO or 5 μmol/L MYCMI-7. Right, flow cytometry analysis of normal REFs treated with DMSO or 5 μmol/L MYCMI-7. **B,** Apoptosis assay using the Cell Death Detection ELISA^plus^ Kit. Left, immortalized Rat1 fibroblasts with different *MYC* status after 24 hours of 5 μmol/L MYCMI-7 treatment, middle panel and right panels, normal REFs and normal peripheral blood lymphocytes, respectively, after 24 and 48 hours of 5 μmol/L MYCMI-7 treatment. *P* values for DMSO versus MYCMI-7 for TGR1, HO15.19, and HOmyc3 cells; 2.76 × 10^−5^, 0.31 and 3.89 × 10^−5^, respectively. *P* values DMSO versus MYCMI-7 for REFs 24 and 48 hours; 0.44 and 0.22, respectively, and for PBLs 24 and 48 hours; 0.19 and 0.18, respectively. **C,** Measurement of viability and apoptosis by Apo-Tox Glo Triplex assay in A375 melanoma cells after 3 days of treatment with MYCMI-7 with the indicated concentrations. **D,** Normal human dermal fibroblasts (NHDF) exposed to indicated concentrations of MYCMI-7 for 72 hours after which cell growth was determined by resazurin assay and by measuring cell number using a cell counter. *P* values resazurin assay DMSO versus MYCMI-7 at 0.5, 1, 5, and 10 μmol/L; 0.0011, 0.00048, 0.00022, and 0.00019, respectively. *P* values cell number DMSO versus MYCMI-7 at 0.5, 1, 5, and 10 μmol/L; 0.11, 0.85, 8.62 × 10^−5^ and 9.44 × 10^−8^, respectively. **E,** NHDFs treated with 3 μmol/L MYCMI-7 for 72 hours. Viability was measured by trypan blue exclusion assay. *P* value DMSO versus MYCMI-7; 0.96. **F,** Cell growth measured with Cyt62 assay of primary normal human epidermal melanocytes (NHEM) treated for 72 hours with MYCMI-7. **G,** Viability measured by trypan blue exclusion assay of NHEM after treatment for 72 hours with MYCMI-7. *P* value DMSO versus MYCMI-7; 0.44. **A**–**G,** One representative biological experiment out of at three, performed in triplicates is shown. The statistical analysis in **B**–**G** were performed by *t* test. *, *P* < 0.05; **, *P* < 0.01; ***, *P* < 0.001; ns, not significant.

We next studied the effect of MYCMI-7 on the cell cycle utilizing P493–6 cells with regulatable MYC (31). Cells synchronized in G_0_–G_1_ by downregulating MYC with doxycycline were allowed to reenter the cell cycle by doxycycline-withdrawal while treated with MYCMI-7 or DMSO. Compared with DMSO, MYCMI-7–treated cells showed a higher G_1_–S ratio, but also strongly induced cell death, as evidenced by the high proportion of sub-G_1_ cells (Fig. 4A, left). In contrast, MYCMI-7 induced G_1_ arrest but not cell death in normal REFs (Fig. 4A, right). Furthermore, MYCMI-7 induced apoptosis in immortalized TGR1 and *MYC*-reconstituted HO15.19 (HOMyc3), but not in *MYC* knockout HO15.19 Rat1 cells (Fig. 4B, left), demonstrating that MYCMI-7–induced apoptosis is MYC dependent. To investigate whether MYCMI-7 induced apoptosis also in tumor cells, we utilized A375 melanoma cells. Titration using these cells showed that MYCMI-7 reduced viability and inversely induced apoptosis in a dose-dependent manner with an GI_50_ in the nanomolar range (Fig. 4C). Looking at different normal cells, we found that MYCMI-7 did not induce apoptosis in normal REFs or normal human peripheral blood lymphocytes (Fig. 4B, middle and right). Normal human dermal fibroblasts (NHDF) treated with MYCMI-7 for 3 days showed a lower overall cell growth as determined by cell count and resazurin assay (which is a redox indicator that undergoes colorimetric change mediated by dehydrogenase enzymes in metabolically active cells) when normalized to exponentially growing DMSO-treated cultures at low micromolar concentrations, while cell viability was unaffected. This suggests that MYCMI-7 induced growth arrest without killing the cells (Fig. 4D and E). Also, in primary normal human epidermal melanocytes (NHEM), MYCMI-7 reduced metabolic activity, but did not induce cell death (Fig. 4F and G).

In conclusion, although MYCMI-7 affected growth of tumor cells and normal cells at similar concentrations, MYCMI-7 mainly triggered apoptosis in tumor cells but induced growth arrest with maintained viability in normal human and rodent cells.

### MYCMI-7 Does not Induce DNA Damage Signaling at Active Concentrations and Acts Independently of Topoisomerase 2 and p53

Considering the structural resemblance of MYCMI-7 to ellipticine (Fig. 1A; Supplementary Fig. S4A), which has been described as a DNA intercalator and topoisomerase 2 (TOP2) inhibitor (39), we were concerned about possible effects of MYCMI-7 on TOP2 activity and DNA damage. We first investigated whether MYCMI-7 is able to inhibit TOP2A, which is the main TOP2 isoform in cycling cells (40). First, we utilized an *in vitro* TOP2A decatenation assay. TOP2 enzymes decatenate kinetoplast DNA (kDNA) converting a network of interlocking DNA rings into individual rings that can be separated by agarose gel electrophoresis. The TOP2 inhibitor doxorubicin started to inhibit TOP2A activity at 3 μmol/L with complete TOP2A inhibition at 10 µmol/L (Supplementary Fig. S4B and S4C), while the TOP1 inhibitor camptothecin has no effect, as predicted. MYCMI-7 exhibited TOP2A-inhibitory activity at concentrations higher than 10 µmol/L with complete TOP2A inhibition at 100 µmol/L. This is consistent with off-target effects starting to appear in MYC knockout cells at higher concentrations than 10 μmol/L (Supplementary Fig. S3B). Ellipticine had no effect on TOP2A activity at any concentration used (Supplementary Fig. S4B and S4C). This indicated that MYCMI-7 was able to inhibit TOP2A activity *in vitro* above 10 μmol/L.

**FIGURE 5:**
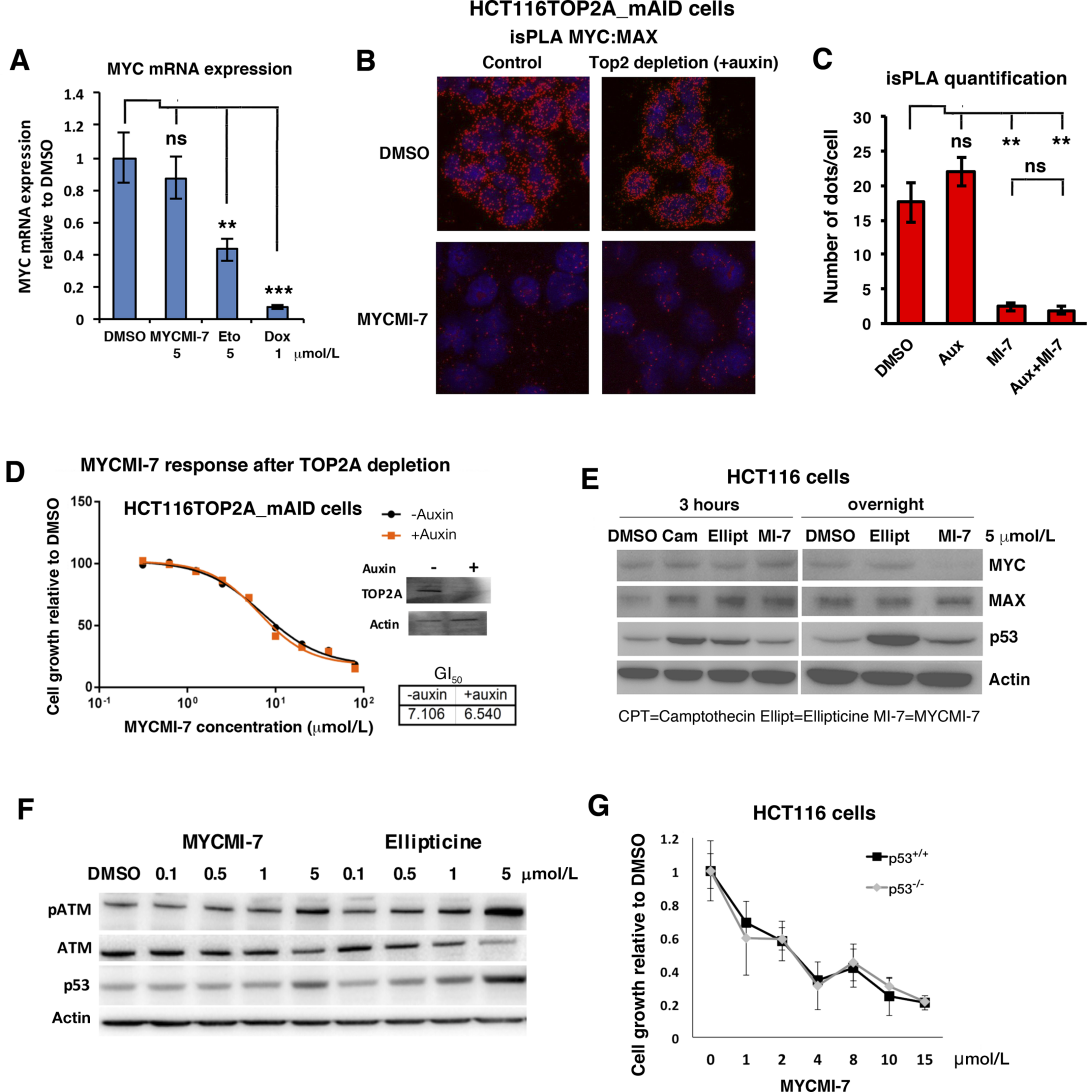
MYCMI-7 does not induce DNA damage response signaling at active concentrations and acts independently of topoisomerase 2 and p53. **A,** Measurement of *MYC* mRNA expression by qRT-PCR after treatment with MYCMI-7 and the topoisomerase 2 inhibitors etoposide (Eto) and doxorubicin (Dox) at the indicated concentrations in MCF7 cells. *P* value for DMSO versus MYCMI-7, etoposide and doxorubicin; 0.35, 0.0036, and 0.00045, respectively, by *t* test. **B**–**D,** Auxin-induced depletion of topoisomerase 2A (TOP2A) in HCT116TOP2A_mAID cells does not affect. **B,** MYC:MAX interactions as measured by isPLA. **C,** Quantification of isPLA dots in **B**. *P* value for DMSO versus auxin, MYCMI-7, and auxin + MYCMI-7; 0.29, 0.026, and 0.025, respectively, by *t* test. *P* value for MYCMI-7 versus auxin + MYCMI-7; 0.80. **D,** Sensitivity to MYCMI-7 as determined by resazurin assay after titration of MYCMI-7 concentrations is the presence and absence of auxin. The Western blot confirms downregulation of TOP2A in response to auxin treatment. GI_50_ values are displayed for MYCMI-7 treatment in the presence and absence of auxin. **E,** Western blot analysis of MYC, MAX, p53, and actin expression after exposure to 5 μmol/L MYCMI-7, ellipticine or camptothecin (CPT) in HCT116 cells. **F,** Western blot analysis of p-ATM, total ATM and p53 expression after exposure to indicated concentrations of MYCMI-7 and ellipticine in A375 cells after 3-hour treatment. **G,***p53* knockout does not affect sensitivity to MYCMI-7 in HCT116 cells as determined by WST-1 assay after titration of MYCMI-7 concentrations as indicated. The cells were harvested 48 hours after treatment.

To address whether the antiproliferative effects of MYCMI-7 in cells could be explained by DNA intercalation and/or TOP2-inhibitory activity, we first compared its ability to downregulate *MYC* mRNA expression (Fig. 5A), which is one well-known effect of DNA intercalators/topoisomerase inhibitors such as doxorubicin and etoposide (41, 42). MYC expression was strongly reduced by 1 μmol/L doxorubicin, and also by 5 μmol/L etoposide in MCF7 cells (Fig. 5A), while 5 μmol/L MYCMI-7 had only minor effect on MYC expression at concentrations where it readily induced growth arrest in the cells in agreement with Fig. 2B, suggesting that MYCMI-7 acts through a distinct mechanism. To investigate whether TOP2A is required for MYCMI-7 activity, we made use of HCT116TOP2A_mAID cells with auxin-regulatable destruction of TOP2A (23, 43). Pretreatment with auxin to deplete TOP2A had no effect on MYCMI-7–induced inhibition of MYC:MAX interactions in the cells as measured by isPLA (Fig. 5B and C), nor did auxin treatment of HCT116TOP2A_mAID cells or siRNA-mediated knockdown of *TOP2A* in MDA-MB-231 breast cancer cells affect the growth-inhibitory effect of MYCMI-7 (Fig. 5D; Supplementary Fig. S4D), arguing against a mechanistic involvement of TOP2A in the action of MYCMI-7. Furthermore, in contrast to ellipticine and the TOP1 inhibitor camptothecin, which induced p53 expression as expected, 5 μmol/L MYCMI-7 had no effect on p53 expression early (3 hours) and only a minor increase late (24 hours) after treatment in HCT116 cells and in *MYCN*-amplified Kelly neuroblastoma cells (Fig. 5E; Supplementary Fig. S4E). We also investigated the p53 and DNA damage response (DDR) response in A375 melanoma cells, which are more sensitive to MYCMI-7–induced growth arrest and apoptosis than HCT116 cells (Fig. 4C vs. Fig. 5D). While 5 μmol/L ellipticine strongly induced phosphorylation of ATM in parallel with increased p53 expression, indicative of DDR signaling, treatment with 5 μmol/L MYCMI-7 showed only a slight increase in these markers, and no p53 or p-ATM induction was observed at concentrations of MYCMI-7 that readily induced growth arrest and apoptosis ( and Figs. 4C and 5F). To address whether *p53* is required for the effects of MYCMI-7, we utilized wt and p53-deficient HCT116 cells. Titration of MYCMI-7 in these two cell lines showed no difference with respect to growth response (Fig. 5G). Western blot analysis showed that MYC expression was significantly downregulated upon 5 μmol/L MYCMI-7 treatment both in *p53*-proficient and *p53*-deficient HCT116 cells, although to a somewhat lesser extent in *p53*-deficient cells (Supplementary Fig. S4F and S4G). Ellipticine did not affect MYC expression at 5 μmol/L, but downregulated the MYC level significantly by around 50% at 10 μmol/L in *p53* wt cells, while it was unaffected in *p53*-deficient cells.

In conclusion, although MYCMI-7 can inhibit TOP2A *in vitro* at higher micromolar concentrations, there were very little signs of such effects in cells at relevant concentrations, as evidenced by the lack of DDR signaling, p53 induction, or *MYC* mRNA reduction. Furthermore, the anti-MYC activity of MYCMI-7 was not dependent on TOP2A or p53.

### MYCMI-7 Is a Potent Inhibitor of *Ex Vivo* Growth of Primary Patient-Derived Glioblastoma and AML Tumor Cells

**FIGURE 6:**
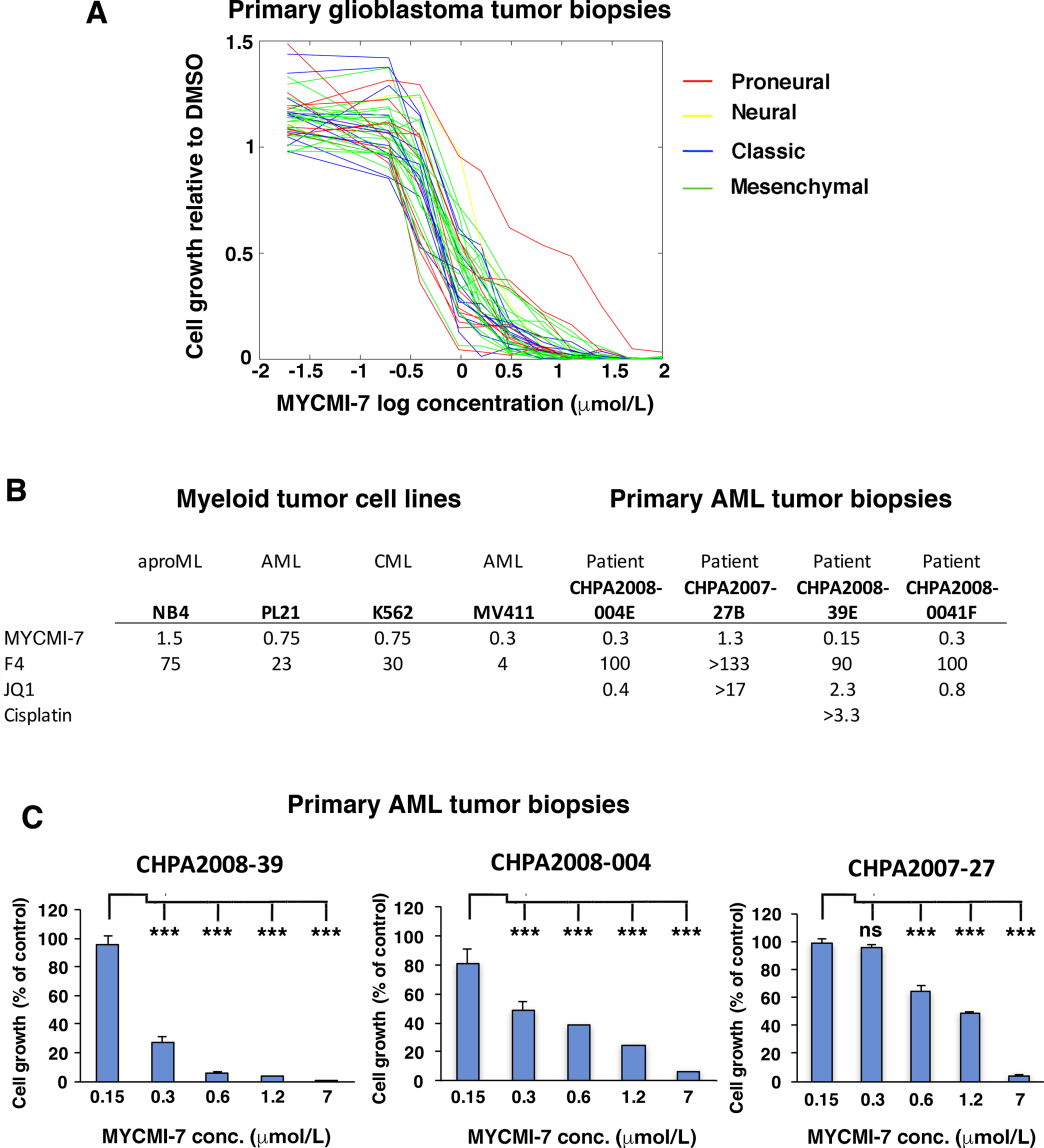
MYCMI-7 is a potent inhibitor of growth of primary patient-derived glioblastoma and AML tumor cells *ex vivo*. **A,** Primary glioblastoma samples from 42 patient biopsies were cultured for 72 hours in presence of MYCMI-7 at indicated doses and cell growth was measured by the resazurin assay. **B,** GI_50_ values for myeloid cell lines and primary AML cells from patient biopsies treated with MYCMI-7 at different concentrations in cell culture and assayed by the WST-1 assay. **C,** Primary AML cells from patient biopsies where cultured for 72 hours in presence of MYCMI-7 at indicated doses cell growth was monitored by the WST-1 assay. One representative biological experiment out of three, performed in triplicates is shown. *, *P* < 0.05; **, *P* < 0.01; ***, *P* < 0.001; ns, not significant. The statistical analysis was performed using *t* test.

Next, we investigated the efficacy of MYCMI-7 using primary patient tumor samples in culture. An *ex vivo* screen of cells derived from primary glioblastoma tumor biopsies of 42 patients, representing different subtypes of glioblastoma (proneural, neural, classical, and mesenchymal; ref. 44), was performed in 2D cultures. MYCMI-7 was very potent with GI_50_ in the submicromolar range, and did not seem to discriminate between subtypes (Fig. 6A; Supplementary Table S1). Furthermore, we found no significant correlation between *MYC* mRNA levels and MYCMI-7 response in this case (Supplementary Table S1). For future studies, it will be important to also collect data on MYC protein levels to include in such correlations. MYCMI-7 also showed potent dose-dependent inhibition of growth of four primary patient-derived AML cell cultures as well as of established AML/CML cell lines, with GI_50_ ranging from 0.15 to 1.3 μmol/L, and were in all cases more efficient than the MYC:MAX inhibitor 10058-F4, the bromodomain inhibitor JQ1, and cisplatin (the latter used in one case; Fig. 6B and C).

### MYCMI-7 Reduces Tumor Burden in a MYC-Driven AML Mouse Model

Encouraged by the results in established tumor cell lines, primary patient-derived tumor cells, and normal cells, we next decided to apply MYCMI-7 *in vivo* in mice. First, we performed a pharmacokinetic study of the behavior of the molecule in healthy mice. Analysis by mass spectrometry of plasma samples collected at 1, 2, 4, and 24 hours after intraperitoneal (i.p.) injection of MYCMI-7 at a concentration of 6.25 mg/kg body weight showed an estimated half-life of 1.5 hours for the compound in plasma (Supplementary Fig. S5A).

**FIGURE 7:**
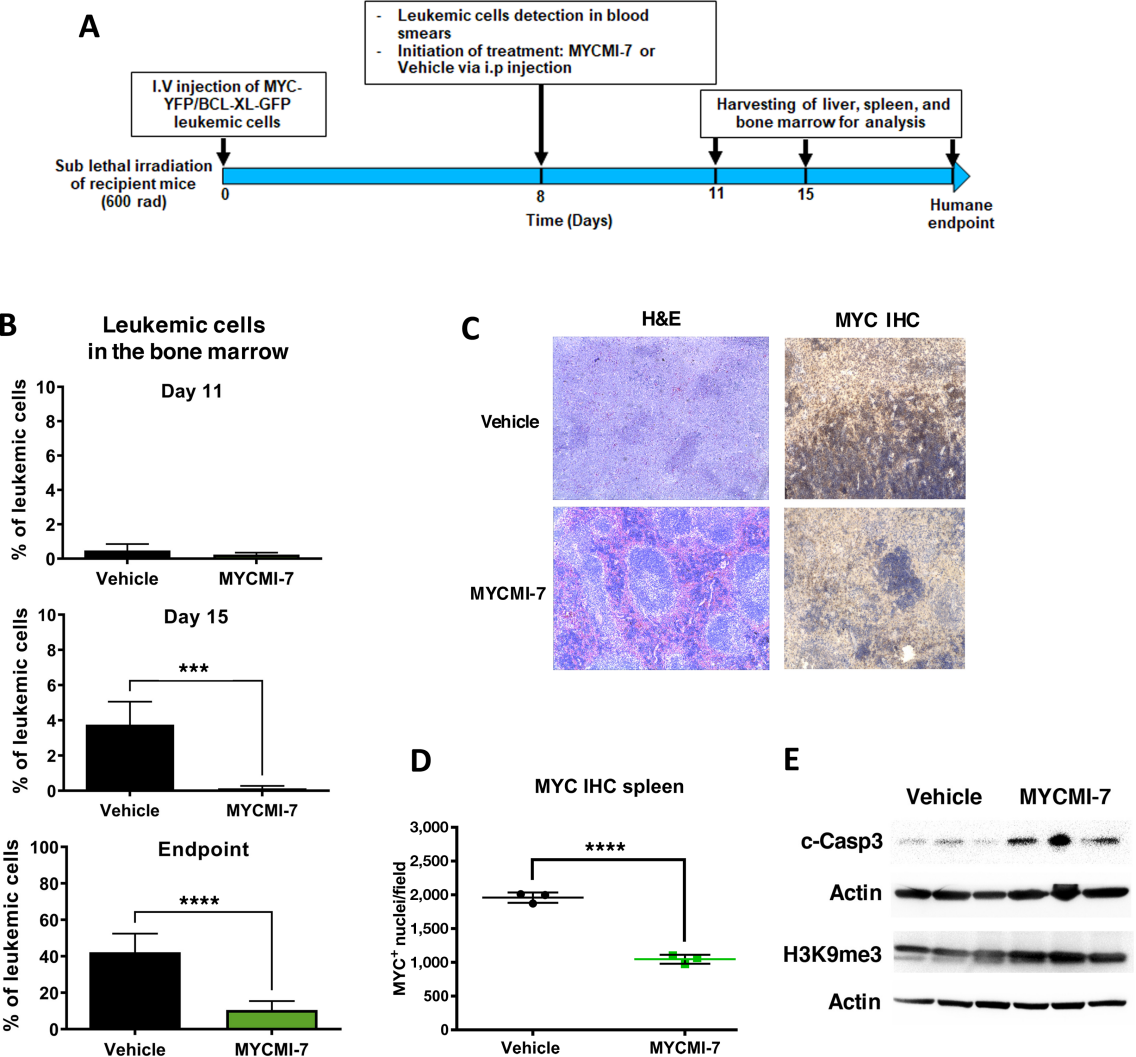
MYCMI-7 inhibits MYC/BCL-XL–driven AML in an allogenic mouse model. **A,** Schematic plan of the experiment. **B,** Flow cytometry analysis of GFP^+^ leukemic blasts in bone marrow on day 11 (*n* = 5), day 15 (*n* = 5), and at the end point after daily intraperitoneal injection of 12.5 mg/kg MYCMI-7 (*n* = 8), compared with vehicle (*n* = 12) mice. *P* values for vehicle versus MYCMI-7 day 15 and endpoint; 0.0003 and <0.0001, respectively. **C,**. H&E and MYC immunohistochemistry (IHC) staining of spleen sections at the endpoint. Images were taken at 10× magnification. **D,** Quantification of MYC IHC staining of spleen sections at the endpoint. *P* value for vehicle versus MYCMI-7; <0.0001. **E,** Western blot analysis of cleaved caspase 3 and H3K9me3 expression in spleens at endpoint after treating mice with MYCMI-7 or vehicle (3 mice from each condition). Actin was used as loading control. At the end points, the last injection was done 3 hours before sacrifice. The statistical analysis was performed using *t* test.

We next investigated potential antitumor effects of MYCMI-7 *in vivo*. First, we utilized a MYC/BCL-X_L_–driven AML mouse tumor model (28, 45). Purified AML cells from spleens of moribund mice placed in culture were highly sensitive to MYCMI-7 treatment, with an GI_50_ less than 1 μmol/L (Supplementary Fig. S5B). After tail vein injection of the purified AML cells into sublethally irradiated syngeneic recipient mice, leukemic blasts were first observed in blood smears at day 8, at which point the mice were treated with 12.5 mg/kg body weight MYCMI-7 daily by intraperitoneal injection. This higher but still tolerated dose was chosen due to the high turnover rate of the compound in plasma (Supplementary Fig. S5A). The mice were then sacrificed according to scheme illustrated in Fig. 7A. GFP-positive leukemic cells were hardly detectable by flow cytometry in the bone marrow at day 11, but reached around 4% at day 15 in vehicle-treated mice, while still undetectable in the MYCMI-7–treated mice, (Fig. 7B, top and middle). At the end point (20 ± 4 days), bone marrow of vehicle-treated mice consisted of around 40% leukemic cells, which was significantly less, at around 10%, in MYCMI-7–treated mice (Fig. 7B, bottom). Similar results were obtained from the spleen (Supplementary Fig. S5C). Interestingly, spleens of MYCMI-7–treated mice retained a more normal histologic spleen structure compared with the collapsed structure of vehicle-treated mice (Fig. 7C). Furthermore, MYC expression was strongly reduced in MYCMI-7- compared with vehicle-treated animals in leukemic cells, as determined by IHC, suggesting that MYCMI-7 reaches its target *in vivo* (Fig. 7C and D). Western blot analysis showed an increased expression of cleaved caspase-3 but also of H3K9me3 in leukemic spleens of mice treated with MYCMI-7 compared with vehicle (Fig. 7E), suggesting concurrent induction of both apoptosis and senescence. Despite this, there was no significant difference in mouse survival between the treatments (Supplementary Fig. S5D). Furthermore, there were no signs of severe side effects of MYCMI-7 treatment; all mice remained healthy and retained their weight over time of the experiment (Supplementary Fig. S5E, see Materials and Methods for mouse safety parameters applied).

In conclusion, MYCMI-7 treatment delayed onset of AML, decreased tumor burden, reduced MYC expression, and induced apoptosis and senescence markers in leukemic cells, but did not improve overall survival. One should, however, bear in mind that this is an extremely aggressive mouse tumor model.

### MYCMI-7 Reduces Tumor Burden and Increases Survival in Xenograft Tumor Models of Basal-Like Breast Cancer and *MYCN*-amplified Neuroblastoma

**FIGURE 8:**
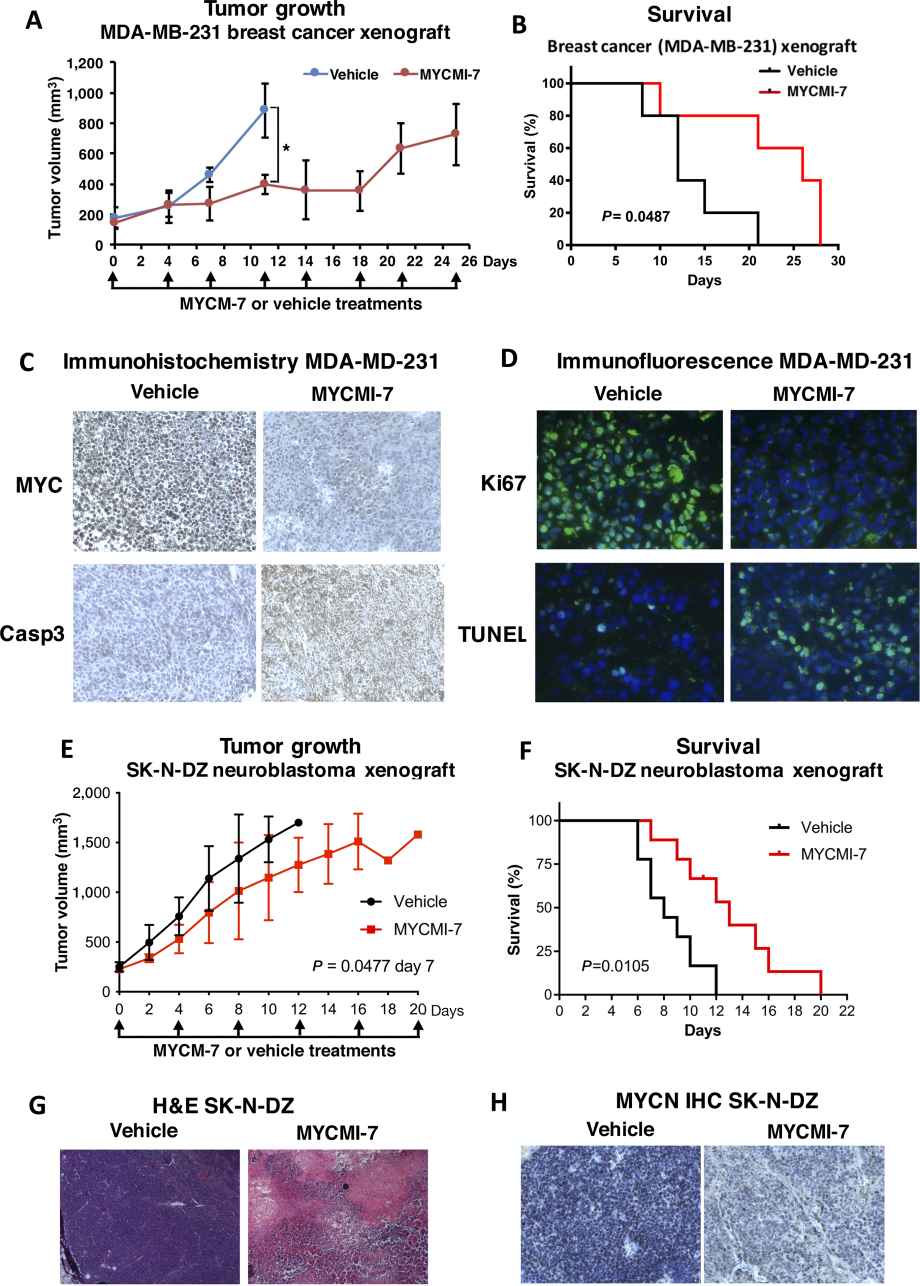
MYCMI-7 inhibits tumor growth and prolongs survival in breast cancer and neuroblastoma xenograft experiments. **A,** Tumor growth of MDA-MB-231 xenografts in mice injected with 6.25 mg/kg MYCMI-7 or vehicle intratumorally twice a week until sacrificed. Tumor growth was monitored by measuring tumor size (mm^3^) every fourth day. *P* value for vehicle versus. MYCMI-7 day 11; 0.030 by *t* test. **B,** Survival curve of the mice in **A** sacrificed when the tumors reached a volume of 1,000 mm^3^. *P* value for vehicle versus MYCMI-7; 0.0487 by log-rank test. **C,** IHC staining for MYC and apoptosis marker caspase 3 in MDA-MB-231 tumor tissue from vehicle- and MYCMI-7 treated animals at end point. **D**, Immunofluorescence staining of proliferation and apoptosis markers Ki67 and TUNEL, respectively, in MDA-MB-231 xenograft tumor tissue from vehicle- and MYCMI-7 treated animals at end point. **E,** MYCN-amplified SK-N-DZ neuroblastoma xenograft mice injected with 6.25 mg/kg MYCMI-7 or vehicle intratumorally twice a week until sacrificed. Tumor growth was monitored by measuring tumor volume (mm^3^) daily. *P* value for vehicle versus MYCMI-7 at day 7 when all mice were still alive; 0.0477 by *t* test. **F,** Survival curve of mice in **E** sacrificed at a tumor volume of 1,500 mm^3^. *P* value vehicle versus MYCMI-7; 0.0105 by log-rank test. **G** and **H,** H&E (**G**) and MYCN IHC (**H**) stainings of SK-N-DZ xenograft tumor tissue from vehicle- and MYCMI-7–treated animals at end point. **A**–**H,** At the end points, the last injection was done 3 hours before sacrifice. Images were taken at 10× or 40× magnification.

We next studied the effects of MYCMI-7 in a xenograft model of the human breast cancer cell line MDA-MB-231. MDA-MB-231 was chosen because it represents basal-like, triple-negative breast cancer, which is a subgroup of breast cancer with frequent *MYC* amplification and/or high MYC pathway activity (2). This subgroup, including MDA-MB-231 cells, has also been reported to be vulnerable to MYC depletion (46–48). The cells were highly sensitive to MYCMI-7 treatment in cell culture (Supplementary Fig. S6A), with a GI_50_ around 1 μmol/L. NOD/SCID mice with established MDA-MB-231 xenograft tumors were treated with 6.25 mg/kg MYCMI-7 or vehicle twice weekly by intratumoral injection. In the solid tumor models with localized disease (in contrast to AML with systemic spread), this route of administration was chosen due to the high turnover rate of the compound in plasma (Supplementary Fig. S5A). After a few days of MYCMI-7 treatment and onwards, tumor growth slowed down considerably compared with vehicle (Fig. 8A), and resulted in a significantly increased survival of the mice (Fig. 8B). Hematoxylin and eosin (H&E) staining of tumor areas showed extensive necrosis/apoptosis in MYCMI-7–treated mice (Supplementary Fig. S6B). Furthermore, IHC staining of tumors revealed reduced expression of MYC and increased caspase-3 expression in response to MYCMI-7, indicative of apoptosis induction (Fig. 8C; Supplementary Fig. S6C and S6D). The latter was also supported by increased TUNEL staining (Fig. 8D; Supplementary Fig. S6E). The tumors were also characterized by reduced proliferation and microvascular density as determined by immunofluorescence staining of Ki67 and CD31 (Fig. 8D; Supplementary Fig. S6F–H), which are both typical characteristics of MYC inhibition *in vivo* (8, 49).

We next evaluated the efficacy of MYCMI-7 in the *MYCN*-amplified SK-N-DZ neuroblastoma xenograft model in NMRI nu/nu mice. After the tumors had reached a volume of around 200 mm^3^, MYCMI-7, or vehicle were administered by intratumoral injection at a dose of 6.25 mg/kg body weight twice a week. MYCMI-7 treatment reduced tumor growth compared with vehicle (Fig. 8E; Supplementary Fig. S6I), resulting in a significantly increased survival (Fig. 8F). H&E staining of tumor tissue showed areas of massive apoptosis and/or necrosis as well as hemorrhage (Fig. 8G). Furthermore, strong reduction in MYCN expression was observed in tumor cells by IHC (Fig. 8H; Supplementary Fig. S6J), again indicating the MYCMI-7 had reached its target in tissues.

Taken together, these results show that MYCMI-7 has the capacity to inhibit growth of breast cancer and neuroblastoma xenograft tumors *in vivo* and to increase survival of mice, as well as to reduce MYC/MYCN expression and tumor cell proliferation, and to trigger apoptosis and necrosis in tumor tissues.

## Discussion

In a cell-based MYC:MAX interaction inhibition screen, we recently identified several active compounds, including MYCMI-6 and MYCMI-7 (8), among which MYCMI-7, which in addition to inhibiting MYC:MAX interaction also downregulates MYC protein levels, is in the focus of this study. Using three cell-based protein interaction assays, split GLuc, isPLA, and co-IP, we found that MYCMI-7 drastically inhibited the MYC:MAX and MYCN:MAX interactions in cells already within 4 hours of treatment at single-digit micromolar concentrations, but did not affect hetero- or homo-dimerization between similar types of transcription factors, thus demonstrating both efficacy and selectivity (Fig. 1). The structurally related compound ellipticine had no effect on either of these interactions. Furthermore, MYCMI-7 treatment rapidly led to reduced association with MYC to target gene promoters. The efficacy of MYCMI-7 toward MYC:MAX in cells was similar to that of MYCMI-6 (8), but clearly higher than what has been reported for other MYC:MAX inhibitors, such as 10058-F4, 10074-G5, #474, KJ-Pyr-9, sAJM589, MYCi361, and MYCi975 (9–11, 50–53).

In biochemical SPR assays, MYCMI-7 was shown to bind directly to the bHLHZip region of MYC with an affinity of approximately 4 μmol/L, which is in good agreement with the cellular GLuc, isPLA and co-IP data (Fig. 1K). By comparison, this is slightly lower affinity than observed for MYCMI-6 (*K*_d_ = 1.6; 8), but at least a magnitude higher than what was reported for 10074-G5, 10058-F4, and #474 (51) using SPR. Using fluorescence polarization competition assay with 10074-G5, Han and colleagues measured a *K*_d_ of 3.6 and 2.5, respectively, for MYCi361 and MYCi975 (10), although one should bear in mind that this is not a direct binding assay and therefore may be difficult to compare.

Subsequent to MYC:MAX inhibition, MYCMI-7 decreased the steady-state levels of MYC protein (but not mRNA), at least in part through increased protein turnover (Fig. 2). The exact mechanism behind this is unclear, but has also been observed with some but not all other MYC:MAX inhibitors (9, 10, 51, 54), as well as after deletion of *MAX* (55), suggesting that dimerization with MAX can stabilize MYC levels. Considering the time difference between MYCMI-7–mediated inhibition of MYC:MAX dimerization and the increased MYC degradation, the latter could also be an indirect result of blocking the MYC–MAX pathway that eventually activates a negative feedback loop resulting downregulation of MYC protein expression. The increased MYC turnover rate observed after MYCMI-7 treatment seemed in part to be dependent on FBXW7, which is a major E3 ubiquitin ligase regulating MYC protein levels by targeting MYC phosphorylated at Thr-58 (32, 33). This was further supported by expression of a Thr-58 → Ala mutant in *MYC* knockout cells, which was partially resistant to MYCMI-7–mediated downregulation of MYC. In contrast, expression of a S62A mutant was downregulated even more strongly by MYCMI-7 than wt MYC. Phosphorylation at Ser-62 has previously been shown to stabilize MYC (56), possibly explaining why loss of this phosphorylation site would make the protein more sensitive to MYCMI-7–mediated destabilization. However, since MYCMI-7 partially stimulated MYC degradation also in FBXW7-deficient cells as well as in cells expressing the T58A mutant, this indicates that also other factors are involved in this process. Analysis of MYC deletion mutants suggested that the C-terminus and the basic DNA-binding region of MYC play a role, but further investigations are required in the future to shed more light on this mechanism. In contrast to our results that did not reveal any difference in MYC Thr58/Ser62 phosphorylation in response to MYCMI-7, Han and colleagues reported that treatment with MYCi361 or MYCi975 induced phosphorylation of MYC at Thr-58 and subsequent degradation of wt MYC but not T58A or S62A MYC mutants (10), suggesting that different MYC:MAX inhibitors can affect MYC turnover in different ways.

MYCMI-7 inhibited cell growth in a MYC-dependent manner, as demonstrated by MYC knockout versus reconstituted Rat1 cells and neuroblastoma cells with or without *MYCN* amplification (Fig. 3). This was further supported by the significant correlation between the levels of MYC mRNA/protein and the response to MYCMI-7 within the NCI-60 tumor cell line panel. In addition, RNA-seq data showed that the MYC and E2F pathways were downregulated, while pathways connected to immune response was upregulated in response to MYCMI-7. The latter is expected considering the role of MYC in suppressing immune surveillance (35, 36), and is consistent with the recently reported effects of the MYC:MAX inhibitors MYCi361 and MYCi975 (10).

The antitumor cell growth efficacy of MYCMI-7 was in the low single-digit micromolar range in most cases and even lower in 3D cultures, which is well in agreement with its efficacy toward MYC:MAX interaction in cells. This is in a similar range as reported for MYCMI-6, certain analogues to 10074-G5, KJ-Pyr-9, sAJM589, MYCi361, and MYCi975, while it is being much more potent in comparison to the “first-generation” MYC:MAX inhibitors, 10058-F4 and 10074-G5 (8–11, 18). The GI_50_ for MYCMI-7 toward patient-derived primary glioblastoma and AML *ex vivo* cultures were in the range of 150 nmol/L–1.3 μmol/L (Fig. 6), demonstrating an excellent efficacy that shows promise for further investigation.

Importantly, while MYCMI-7 induced cell death/apoptosis in tumor cells, it was not cytotoxic to a range of normal primary cells, including human and murine fibroblasts, human melanocytes, and human peripheral blood lymphocytes, where it instead induced G_1_ arrest, indicating that MYCMI-7 is nontoxic to normal cells at active concentrations (Fig. 4). It has been observed previously that inhibition of endogenous MYC by Omomyc in mouse models induced apoptosis in tumor cells, but only a reduction in proliferation in normal tissues, which was reversible upon MYC reactivation (57). This difference in response to MYC inhibition in malignant and normal cells has been described as “oncogene addiction” (1, 4).

Because MYCMI-7 has a structural resemblance to ellipticine, which has been described as a DNA intercalator and TOP2 inhibitor (39), we were concerned that MYCMI-7 might have similar activities. MYCMI-7, in contrast to doxorubicin, did not inhibit TOP2A activity until reaching concentrations above 10 μmol/L (Supplementary Fig. S4). However, when investigating cellular responses to MYCMI-7, it clearly differed from DNA interactors and/or TOP1/2 inhibitors such as ellipticin, doxorubicin, etoposide, camptothecin. In contrast to the latter drugs, MYCMI-7 did neither downregulate *MYC* mRNA expression, nor induce p53 or phosphorylation of ATM at active concentrations, suggesting that it is not a potent inducer of DNA damage responses (Fig. 5). Furthermore, the growth-inhibitory/apoptotic activity of MYCMI-7 was not dependent on p53 or TOP2A activity, suggesting that the anti-MYC activity of MYCMI-7 is not related to DNA intercalation, TOP2A inhibition, or DDR signaling.

The *in vivo* potential of MYCMI-7 was investigated in three MYC-driven mouse tumor models: AML, breast cancer, and MYCN-amplified neuroblastoma. Despite having a half-life in plasma of approximately 1.5 hours, MYCMI-7 treatment reduced tumor volume significantly in all three models and increased overall survival in two of three models (Figs. 7, 8), with exception of MYC/BCL-XL–driven AML. The latter is surprising considering the clear effect of MYCMI-7 on tumor load. One should remember that this is an extremely aggressive model (28, 45), and we speculate that the rapid expansion of leukemic cells in several organs together with the massive apoptosis (and senescence) of such cells after MYCMI-7 treatment may cause systems collapse leading to death despite effects of treatment. In all three tumor models, MYCMI-7 treatment led to reduced MYC or MYCN expression in tumor tissue. It also led to massive induction of apoptosis/necrosis and reduced tumor cell proliferation, which are typical outcomes when MYC is inactivated in transgenic mouse tumor models with inducible MYC (49, 57–59), as well as after treatment with MYCMI-6, 10008-F4, and with Mycro3 (8, 54, 60). In the solid tumors, there was a reduction in microvascularity and signs of increased hemorrhage, which might reflect collapse of tumor vasculature previously observed in the Omomyc tumor model after MYC inhibition (49) and after MYCMI-6 treatment in a *MYCN*-amplified neuroblastoma xenograft mouse model (8). Inhibition of tumor cell growth *in vivo* using mouse tumor models has been reported previously for the MYC:MAX inhibitors MYCMI-6, 10058-F4, KJ-Pyr-9, KSI-3716, Mycro3, MYCi361, and MYCi975 (8, 10–12, 54, 60). In these studies, MYCMI-6 and Mycro3 were shown to reduce activity or expression of MYC in tumor tissue. Importantly, MYCMI-7 treatment was well tolerated by the mice, which did not lose weight after daily systemic treatment of 12.5 mg/kg body weight, and there were no signs of other severe side effects.

In summary, we show that MYCMI-7 is a direct MYC-binding compound that potently and selectively inhibits MYC:MAX interaction and MYC-mediated gene regulation and tumor cell growth in a MYC-dependent manner in culture and *in vivo*, while sparing normal cells. MYCMI-7 shows similar potency and selectivity as MYCMI-6 *in vitro*, in cells and *in vivo*, but in contrast to MYCMI-6, it also reduces MYC protein stability, and therefore potentially has higher therapeutic efficacy. MYCMI-7 together with other MYC inhibitors thereby contributes to a “tool box” with different types of MYC inhibitors that can used not only in MYC research but most importantly can be developed further and potentially could be utilized for treatment of different types of MYC-driven cancers alone or in combination. Considering the significant discrimination of MYCMI-7 between *MYCN*-amplified and nonamplified neuroblastoma and between the NCI-60 cancer cell lines with “high” versus “low” MYC expression, we anticipate that both *MYC* amplification and elevated MYC expression, can be used as biomarkers for identification of patients with cancer likely to benefit from MYC inhibitor treatment. *MYC* family gene amplification is frequent in neuroblastoma, ovarian cancer, basal-like breast cancer, lung, colon, pancreatic, and other cancers, MYC translocation is observed in Burkitt's lymphoma and some other lymphomas, and deregulated MYC expression is frequent in many different types of cancer (2). Hopefully, MYCMI-7 together with other MYC inhibitors can pave the way for the development of clinically relevant anti-MYC therapy for these and other types of cancer in the future.

## Supplementary Material

Supplementary Materials and Methods, Figures 1-6 Supplementary Figure 1. MYCMI-7 selectively inhibits the MYC:MAX interaction in cells and blocks MYC's association with chromatin. Supplementary Figure 2. MYCMI-7 downregulates MYC and MYCN protein expression. Supplementary Figure 3. MYCMI-7 reduces tumor cell growth/viability of MYC-driven tumor cells and inhibits oncogenic transformation in 2D and 3D cultures. Supplementary Figure 4. Effects of MYCMI-7 is relation to TOP2A activity in vitro and in cells. Supplementary Figure 5. Treatment of MYC/BCL-XL-driven acute myeloid leukemia with MYCMI-7. Supplementary Figure 6. MYCMI-7 inhibits growth of MDA-MB-231 breast cancer cell and SK-N-DZ MYCN-amplified neuroblastoma cell xenografts in vivo.
