# Supplementary material for: MYCMI-7: A Small MYC-Binding Compound that Inhibits MYC: MAX Interaction and Tumor Growth in a MYC-Dependent Manner
Source: Cancer Res Commun. 2022 Mar 31;2(3):182–201. doi: 10.1158/2767-9764.CRC-21-0019 (PMC9980915; doi:10.1158/2767-9764.CRC-21-0019)
Supplement: Supplementary Materials and Methods, Figures 1-6 — Supplementary Figure 1. MYCMI-7 selectively inhibits the MYC:MAX interaction in cells and blocks MYC's association with chromatin. Supplementary Figure 2. MYCMI-7 downregulates MYC and MYCN protein expression. Supplementary Figure 3. MYCMI-7 reduces tumor cell growth/viability of MYC-driven tumor cells and inhibits oncogenic transformation in 2D and 3D cultures. Supplementary Figure 4. Effects of MYCMI-7 is relation to TOP2A activity in vitro and in cells. Supplementary Figure 5. Treatment of MYC/BCL-XL-driven acute myeloid leukemia with MYCMI-7. Supplementary Figure 6. MYCMI-7 inhibits growth of MDA-MB-231 breast cancer cell and SK-N-DZ MYCN-amplified neuroblastoma cell xenografts in vivo. [file crc-21-0019-s01.pdf]

## Supplementary Information

### Supplementary Materials & Methods

### Cell culture and transfection

HeLa, MCF7, MDA-MB-231, U2OS, HCT116 (and sublines), SK-N-F1, SK-N-RA, SK-N-AS, A375, COS-7, Rat1 fibroblasts (Tgr1, H015.19 and H0MYC3), primary rat embryonic fibroblasts (REFs) were maintained in DMEM supplemented with 10 % fetal bovine serum (FBS) and penicillin/streptomycin.. Kelly, IMR-32, SK-N-DZ, P493-6, Daudi, CA46 and Ramos were kept in RPMI supplemented with 5 % FBS and antibiotics. In addition, the medium for H015.19 and primary REFs contained 1% sodium pyruvate. All cells were mycoplasma free and kept at 37°C and 5% $CO_2$. To turn off expression of the MYC gene in P493-6 cells, a final concentration of 1 μg/ml doxycycline (Sigma) was added to the culture medium for 24 hours (1). Transfection of HeLa cells was performed using Lipofectamine 2000 reagent (Invitrogen) according to manufacturer's instructions. For transfection of HEK293, U2OS and REF cells, FuGene6 (ROCHE) was used in accordance with manufacturer's recommendations. The proteasome inhibitor Z-leu-leu-leu-H aldehyde (MG115) (Peptides International) was used at 40 μM concentration.

For anchorage-independent growth assay, cells were suspended in 250 μl 0.35% SeaPlaque agarose (InVitro) and seeded into 24 well plates which had previously been coated with a bottom layer of 250 μl 0.7% agarose. After 16 days, the colonies were stained with 100 μg/ml MTT (Sigma) overnight and colonies were counted. For foci formation assays, $3x10^4$ REFs were seeded into 6 well plates. The following day the cells were transfected with the indicated plasmids (0.3 μg of each, a total of 0.6 μg per well). After another 24 hours compounds were added. The medium was changed every 2-3 days. When the cells had grown into confluency, medium containing 4% serum was added. After 14 days the number of foci was scored.

### Cell growth and viability assays

Cell viability by the Cyt60 assay was carried out as in manufacturers' protocol. For apoptosis assays, cells were seeded into 96 well plates, treated with compounds, and 24 hours later the cells were harvested and 2500 cells per analysis were used in the Cell Death Detection ELISA$^{plus}$

kit (ROCHE) according to the manufacturer's recommendations. To evaluate cell viability in combination with apoptosis (caspase activation), the Apo-Tox Glo Triplex assay (Promega) was used. Fluorescence detection of viability (400Ex/505Em) and luminescence measurement of caspase activation was obtained in a 96-well plate format., according to the manufacturer's instructions.

**Viability measurements in glioblastoma initiating cell cultures**

The adherent human glioblastoma initiating cell cultures (2) were seeded on laminin-coated 384-well microplates (BD Falcon Optilux #353962). The optimal cell number for each culture was determined to ensure that it was in growth phase at the end of the assay (~70% confluency which resulted in between 2000 – 4000 cells/well). Cell cultures (45 for screen 2 and 24/29 for screen 3) were plated one day prior to treatment using a Multidrop plate dispenser (ThermoScientific). Drugs were transferred using an ECHO550 non-contact liquid dispenser (Labcyte, USA) to a 384 standard dispensing plate. The substances were subsequently diluted in medium and lifted with a 384 head and a Janus MTD liquid dispenser (PerkinElmer, USA) to the cell plates. The screening experiment was assayed for viability using resazurin (R7017, Sigma Aldrich) after 72 hours of treatment as previously described (3), and detected by a fluorescent plate reader (EnVision multilabel reader, PerkinElmer) at excitation and emission wavelengths of 535/595 nm. As a negative control, DMSO vehicle was added at 4 different concentrations (16 wells/plate) and doxorubicin was used as a positive control (one 11-dose response curve on each plate). Each treatment dose was mapped to their individual exact DMSO concentration for normalization. The screening protocol included a dose range of 11 doses ranging from 20 nM - 100 µM (screen 2) and 0.5 nM - 50 µM (screen 3). All cell cultures are profiled and mapped to a specific glioma subgroup (Voichita Marinescu and Sven Nelander, unpublished), included in the analysis pipeline. Cells were screened in batches of 6-12 in an effort to optimize mixing of subgroups.

**Plasmids**

Zip-hGLuc(1) and Zip-hGLuc(2) kindly provided by S. Michnick (University of Montreal, Montreal) were used to create MYC-Luc2, NMYC-Luc2 and MAX-Luc1 by replacing the existing Zip-gene (GCN4) with full length MYC, MYCN or MAX cDNA. Vectors where the Zip-protein was cut out but not replaced (Luc1 and Luc2) was also created (details available on request).

Other plasmids used were pCMV-LUC, pLXSMYCN, pEVBJ-RAS, pCDNA3-flag-MYC wt, T58A, S62A, and pCDNA3. N-terminal 6xHis-tagged constructs MYCbHLHZip and MAXbHLHZip were cloned into pET28a.

**Antibodies.** Antibodies used for isPLA for cell cultures were C-33 α-MYC or B8.4.B α-MYCN combined with C-17 α-MAX or H-50 α-FRA1 (all Santa Cruz Biotechnology) combined with 2315S α-JUN (Cell Signaling Technology), or control DBD α-Gal4 antibody (Santa Cruz), all diluted 1:50. Immunoprecipitation of MYC was performed with α-MYC N262 (Santa Cruz biotechnology) or α-MYC Y69 (Abcam) antibodies. For ChIP, α-MYC N262 (Santa Cruz biotechnology) was used. The following antibodies were used for western blot: α-MYC N262, α-MYC 9E10, α-MYC C-33, α-NMYC (d46-507), α-Cyclin E HE12 (all Santa Cruz), α-MYC Y69 (Abcam), α-P-T58/S62 MYC (Cell Signaling), α-MAX 101271 (Abcam), α-Actin (A2228; Sigma-Aldrich or AC-15, Sigma). For staining of tumor tissue sections, the Ki67 Ab 16667 (Abcam, dilution 1/500), the CD31 Ab 553370 (BD Pharmingen, dilution 1/200), MYC Ab Y69 (Abcam, dilution 1/250), MYCN Ab B8.4.B (Santa Cruz biotechnology, dilution 1/250), Casp3 Ab D175 (Cell Signaling, dilution 1/250) were used.

**Primers for RT-qPCR**

The following forward (FW) and reverse (REV) sequences of human primers were used for RT-qPCR:

GAPDH FW: ACATCGCTCAGACACCATG, REV: TGTAGTTGAGGTCAATGAAGGG,
ODC1 FW: TCTGCTTGATATTGGCGGTG, REV: GGCTCAGCTATGATTCTCACTC,
CR2 FW: GGGTTTTCTTGGCTCTCGTC, REV: CCTTATCACGGTACCAACAGC,
RGS16 FW: CTGCGATACTGGGAGTACTGG, REV: CCACCCCAGCACATCTTC
CAMKV FW: TGGCTGGTGACTATGAGTTTG, REV: CAGCATTGCCAGAAATCCAC
Nucelolin FW: AGGTGACCCCAAGAAAATGG, REV: AGCCTTCTTGCCTTTCTTCTG

**Recombinant proteins**. Recombinant proteins containing His-tagged N-termini were over-expressed in *E. coli BL21* (DE3) bacteria (Stratagene) at 37 °C in 2XTY, or LB, media with kanamycin, and were purified on a Ni-NTA (Qiagen) affinity bench column, or using a HisTrap HP column (5 mL) with an ÄKTA system. The purifications were carried out according to

manufacturer instructions. All the proteins were dialyzed against PBS, pH 7, at 5 °C overnight. The purity of the proteins was confirmed by SDS-PAGE analysis and Mass Spectrometry analysis.

**_Gaussia_ luciferase protein fragment complementation assay (GLuc)**

The Gaussia luciferase protein fragment complementation assay has been described elsewhere (4, 5). 0.2-0.4 μg of each GLuc-construct together with 0.05 μg pCMV-LUC (firefly luciferase) were used per transfection in 12 well plates. 24 hours later cells were treated with compound or DMSO. After another 17 hours, the cells were harvested and lyzed in passive lysis buffer (Promega) supplemented with complete protease inhibitor (Roche). After 60 min incubation at room temperature 20 μM D-luciferin was added (substrate of Firefly luciferase) and luminescence was measured in a Lumat LB9501 (Berthold) or OmegaFluostar (BMG Labtech) luminometer. Directly after, *Gaussia* luciferase substrate Coelenterazine (Promega) was added to a final concentration of 20 μM in a mixture with “Stop n’ Glow” (Promega) and the luciferase activity was measured. The ratio between *Gaussia* and Firefly luciferase values were calculated and normalized to DMSO-treated control cells.

**Surface plasmon resonance (SPR).** The SPR experiments were performed at 25 °C using a Biacore T200 (GE Healthcare) instrument kindly provided by SciLifeLab Solna. An amino coupling procedure was used to immobilize protein on a CM5 sensor chip (GE Healthcare). Sensorgrams were generated by subtraction of the reference (blank immobilized) surface. The MYC SPR assay was carried out as described above with MYCbHLHZip immobilized to a level of 800-1000 RU. For kinetic binding experiments, a Langmuir 1:1 binding event was applied using the Biacore T200 Evaluation Software 2.0 (GE Healthcare) to determine association ($k_a$) and dissociation ($k_d$) constants of the compound and to calculate affinity ($K_D$) by the formula; $K_D=k_d/k_a$. Binding responses from equilibrium binding experiments were plotted against compound concentration and $K_D$ values were determined at 50% of the theoretical Rmax with the formula Rmax = (MW analyte/ MW ligand) x immobilized ligand level on the chip (RU) x stoichiometry (1:1). Same coupling method described above was used to immobilize the control proteins. MAX was immobilized to approximately 2000 RU.

**Flow cytometry**

P493-6 cells were synchronized using 1 μg/ml doxycycline. The cells were washed three times with culture medium and incubated with or without compound for another 48 hours before harvest. REFs were seeded out in 6 well plates 24 hours prior to indicate treatment and harvested by trypsinization after 48 hours of treatment. Cells were stained with propidium iodide (Sigma) and $1x10^4$ cells per treatment were analyzed using a FACScan and CellQuestPro (Becton Dickinson). The cell cycle distribution analysis was performed using the ModFit LT 3.1 software (Verity software House)

**Immunohistochemistry and immunofluorescence**. Apoptosis was visualized at the single-cell level on tumor cryosections using the TUNEL method using the *In Situ* Cell Death Detection Fluorescein- Roche kit and analyzed by fluorescence microscopy. Briefly, tumor cryosections were fixed in 4% paraformaldyde (PFA) and the assay was performed following the instructions from the manufacturer. Negative controls were run using the reagent without the TdT enzyme. Samples were mounted using Vectashield mounting medium (Vector) and DAPI was used as nuclear counterstaining. Cell proliferation and microvascular density (MVD) were evaluated through Ki67 and CD31staining, respectively, on tumor cryosections and detected by immunofluoresence. Visualization and image acquisition was done in a Zeiss microscope, and for panoramic views by a Vectra imaging system. For quantification, areas with positive TUNEL and Ki67 staining were measured using Image J software. For the analysis of MVD, the number of vessel structures per microscopic field was calculated.

**Top2 decatenation assay**

Recombinant human Top2α (15 nM) was incubated with 80ng catenated kinetoplast DNA (Topogen, TG2013) in Top2 reaction buffer (50 mM Tris pH8, 150 mM NaCl, 10 mM $MgCl_2$, 0.5 mM DTT, 30 µg/ml BSA) with 1-100 µM of either doxorubicin, MYCMI-7, ellipticine or camptothecin for 10 minutes at 37°C. Drug dilutions were made in a DMSO/$H_2O$ solution so that a final concentration of 1% DMSO was consistent across all samples. The reaction was stopped with the addition of SDS to a final concentration of 1% and the Top2α was digested by Proteinase K at 55°C for 10 minutes. DNA was purified by phenol:chloroform extraction before running on a 1% agarose gel either containing or post-stained with 0.5µg/ml ethidium bromide. The decatenated products were quantified, normalised to the Top2α + DMSO control and plotted using GraphPad Prism.

**Pharmacokinetics**

MYCMI-7 was dissolved to 12.5 mg/mL in DMSO:glycerol 9:1 and was further diluted to 1.25 mg/mL in 9% DMSO:saline before administration to mice by intraperitoneal (i.p.) injection. Blood and brains were collected from three to four mice per group at 0, 1, 2, 4 and 24 hours following injection of 6.25 mg/kg ToM. Plasma samples were extracted using protein precipitation. Brain samples were extracted by homogenization in acetonitrile:DMSO, 95:5 using a gentleMACS Dissociater and gentleMACS™ M Tubes. The protein in the mouse plasma samples was removed by protein precipitation with acetonitrile containing the internal standard. After protein precipitation the study samples were centrifuged, and the resulting supernatant was analyzed. The acetonitrile:DMSO from the brain sample homogenates were spiked with the internal standard and then analyzed. All samples were analyzed by first separating them by reversed phase gradient LC and subsequently detecting them using positive electrospray ionization and multiple reaction monitoring (MRM).


1. Schuhmacher M*, et al.* (1999) Control of cell growth by c-Myc in the absence of cell division. *Curr Biol* 9(21):1255-1258.
2. Pollard SM*, et al.* (2009) Glioma Stem Cell Lines Expanded in Adherent Culture Have Tumor-Specific Phenotypes and Are Suitable for Chemical and Genetic Screens. *Cell Stem Cell* 4(6):568-580.
3. Nociari MM, Shalev A, Benias P, & Russo C (1998) A novel one-step, highly sensitive fluorometric assay to evaluate cell-mediated cytotoxicity. *J Immunol Methods* 213(2):157-167.
4. Castell A*, et al.* (2018) A selective high affinity MYC-binding compound inhibits MYC:MAX interaction and MYC-dependent tumor cell proliferation. *Sci Rep* 8(1):10064.
5. Remy I & Michnick SW (2006) A highly sensitive protein-protein interaction assay based on Gaussia luciferase. *Nat Methods* 3(12):977-979.

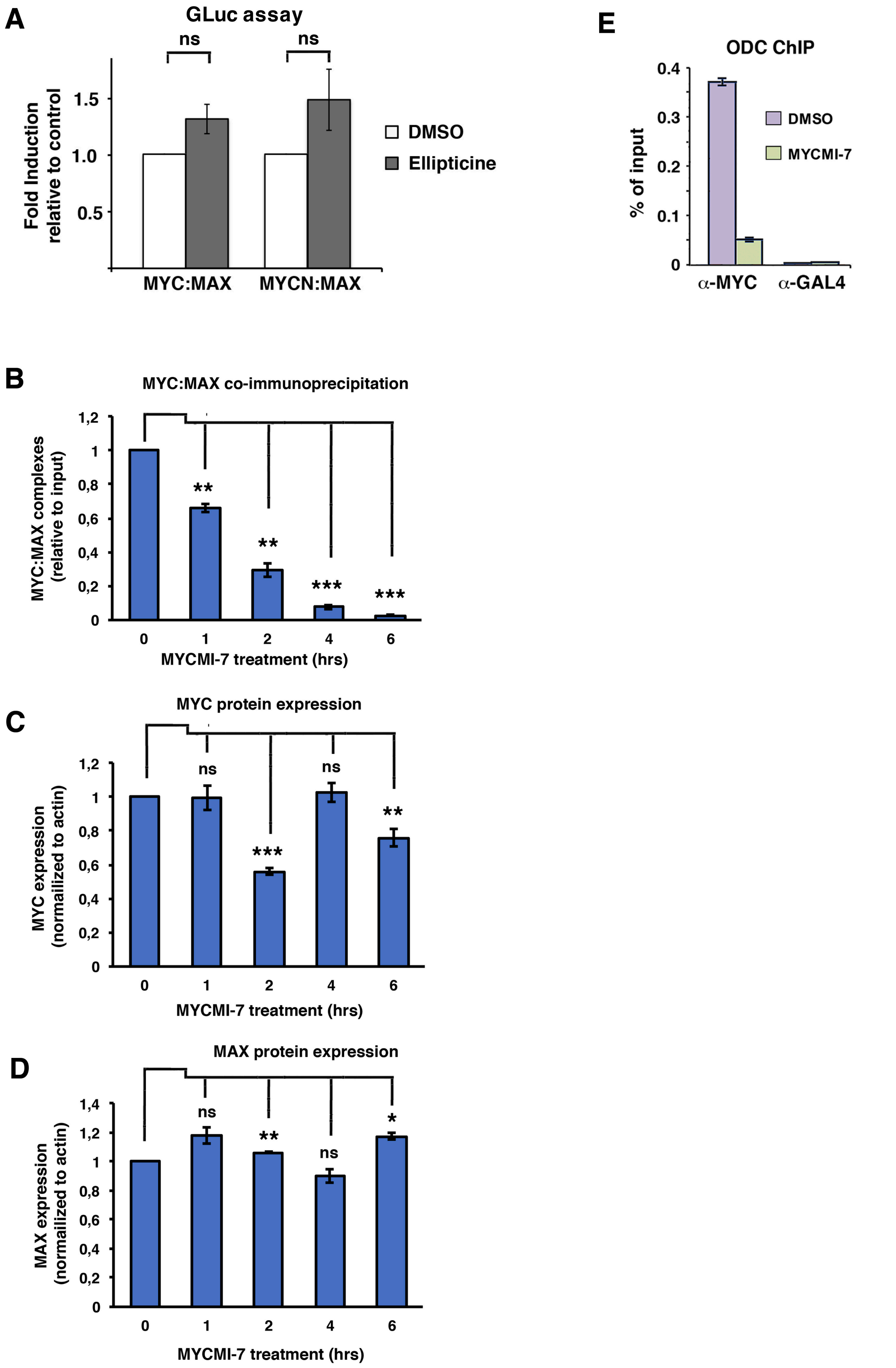
A
GLuc assay
ns
ns
Fold Induction relative to control
1.5
1.0
0.5
DMSO
Ellipticine
MYC:MAX
MYCN:MAX
E
ODC ChIP
0.4
0.3
0.2
0.1
0
% of input
DMSO
MYCMI-7
α-MYC
α-GAL4
B
MYC:MAX co-immunoprecipitation
MYC:MAX complexes (relative to input)
1,2
1
0,8
0,6
0,4
0,2
0
**
**
***
***
0
1
2
4
6
MYCMI-7 treatment (hrs)
C
MYC protein expression
MYC expression (normailized to actin)
ns
ns
***
**
MYCMI-7 treatment (hrs)
D
MAX protein expression
MAX expression (normailized to actin)
1,4
ns
**
ns
*
MYCMI-7 treatment (hrs)

**Supplementary Figure 1.** MYCMI-7 selectively inhibits the MYC:MAX interaction in cells and blocks MYC's association with chromatin. A) Ellipticine does not reduce MYC:MAX or MYCN:MAX interactions. MYC:MAX and MYCN:MAX GLuc assays after treatment with 5 μM ellipticine in HeLa cells as described in the legend to Fig. 1. p-values DMSO vs. ellipticine for MYC:MAX and MYCN:MAX; 0.855 and 0.185, respectively. B) Quantification of MYC:MAX co-immunoprecipitation, C) of MYC and D) of MAX input (Fig. 1H) normalized to actin expression after MYCMI-7 treatment in MCF7 cells at indicated time points. B) p-values co-immunoprecipitation MYC:MAX, DMSO vs. MYCMI-7 at 1, 2, 4, and 6 hrs; 0.0034, 0.0025, 0.0002 and $9.47x10^{-6}$, respectively. C) p-values MYC input, DMSO vs. MYCMI-7 at 1, 2, 4, and 6 hrs; 0.152, $4.15x10^{-6}$, 0.051 and 0.0011, respectively. D) p-values MAX input, DMSO vs. MYCMI-7 at 1, 2, 4, and 6 hrs; 0.100, 0.0021, 0.287 and 0.020, respectively. The statistical analysis in A-D) were performed by t-test. E) Chromatin immunoprecipitation (ChIP) of MYC at the ODC1 target gene promoter after treatment of U-937 cells with 5 μM MYCMI-7 for 6 hours. GAL4 antibodies were used as negative controls.

*= p<0.05, **=p<0.01, ***=p<0.001, ns=not significant.

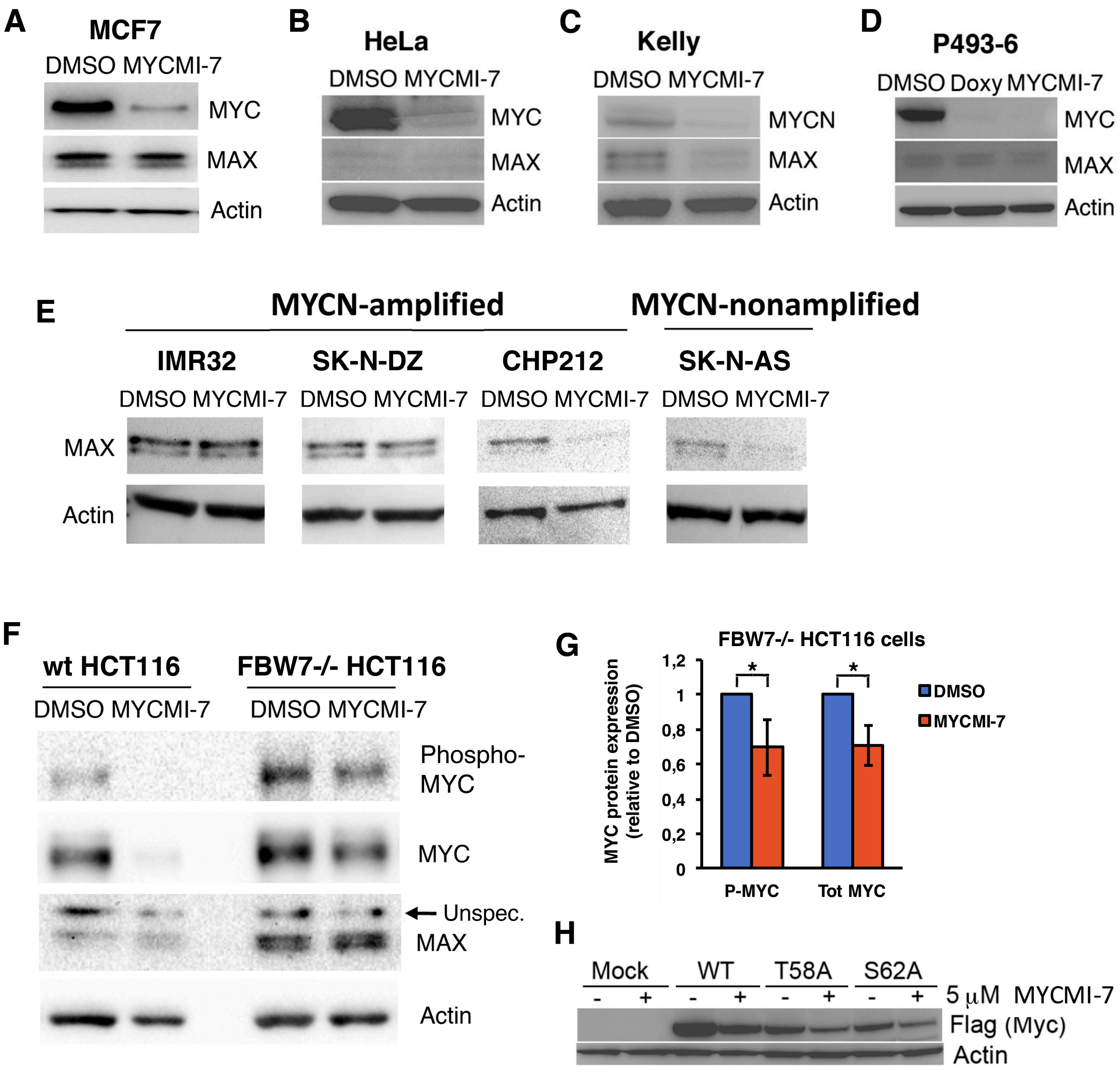
A
MCF7
DMSO MYCMI-7
MYC
MAX
Actin
B
HeLa
DMSO MYCMI-7
MYC
MAX
Actin
C
Kelly
DMSO MYCMI-7
MYCN
MAX
Actin
D
P493-6
DMSO Doxy MYCMI-7
MYC
MAX
Actin
E
MYCN-amplified
MYCN-nonamplified
IMR32
SK-N-DZ
CHP212
SK-N-AS
DMSO MYCMI-7
MAX
Actin
F
wt HCT116
FBW7-/- HCT116
DMSO MYCMI-7
Phospho-MYC
MYC
Unspec.
MAX
Actin
G
FBW7-/- HCT116 cells
MYC protein expression (relative to DMSO)
1,2
1
0,8
0,6
0,4
0,2
0
P-MYC
Tot MYC
DMSO
MYCMI-7
H
Mock
WT
T58A
S62A
5 µM MYCMI-7
Flag (Myc)
Actin

**Supplementary Figure 2.** MYCMI-7 downregulates MYC and MYCN protein expression. A-D) Expression MYC, MYCN and MAX proteins in A) MCF7 cells, B) HeLa cells, C) Kelly MYCN-amplified neuroblastoma cells, D) P493-6 cells harboring a doxycycline (Doxy)-regulated MYC gene. A-D) cells were treated with 5 μM MYCMI-7 for 17 hours and subjected to western blot analysis. In D), cells were treated with 1μg/ml doxycycline (Doxy) (to turn MYC expression off) or without Doxy (to keep MYC expression on). E) Expression of MAX and actin in indicated neuroblastoma cells treated with DMSO or 5 μM MYCMI-7 overnight as determined by western blot analysis. F) Expression of phosphorylated and total MYC after 5 μM MYCMI-7 treatment for 17 hrs in wt or FBXW7-/- HCT116 cells. Antibodies recognizing P-T58/S62 or total MYC were used in western blot analysis. G) Quantification of phosphorylated and total MYC protein expression in HCT116 FBXW7-/- cells in F) normalized to actin expression. p-values DMSO vs. MYCMI-7 for phosphorylated and total MYC; 0.012 and 0.015, respectively. The statistical analysis was performed by t-test. H) U2OS cells were transfected with vectors containing wt, T58A or S62A MYC for 24 hrs, after which the cells were treated with 5 μM MYCMI-7 overnight. The steady state levels of MYC and MYC mutant proteins were analyzed by western blot. Note that this experiment was carried out in the presence of the proteasome inhibitor MG115 to avoid differences in steady state levels of MYC.

*= $p<0.05$, **=$p<0.01$, ***=$p<0.001$, ns=not significant.

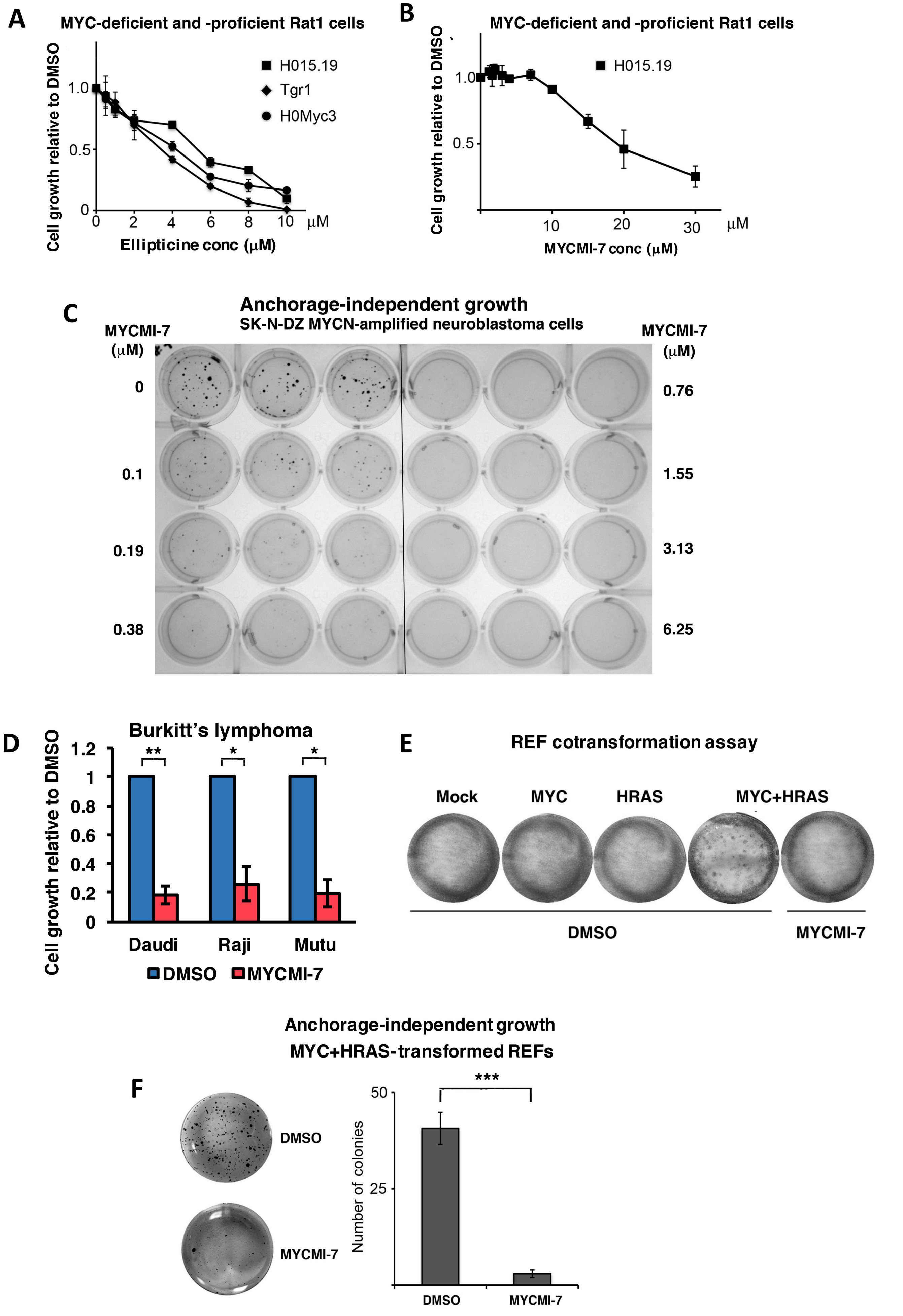
A
MYC-deficient and -proficient Rat1 cells
Cell growth relative to DMSO
H015.19
Tgr1
H0Myc3
Ellipticine conc (µM)
B
MYC-deficient and -proficient Rat1 cells
Cell growth relative to DMSO
H015.19
MYCMI-7 conc (µM)
C
Anchorage-independent growth
SK-N-DZ MYCN-amplified neuroblastoma cells
MYCMI-7 (µM)
0
0.1
0.19
0.38
0.76
1.55
3.13
6.25
D
Burkitt's lymphoma
Cell growth relative to DMSO
Daudi
Raji
Mutu
DMSO
MYCMI-7
E
REF cotransformation assay
Mock
MYC
HRAS
MYC+HRAS
DMSO
MYCMI-7
Anchorage-independent growth
MYC+HRAS-transformed REFs
F
DMSO
MYCMI-7
Number of colonies
***

**Supplementary Figure 3.** MYCMI-7 reduces tumor cell growth/viability of MYC-driven tumor cells and inhibits oncogenic transformation in 2D and 3D cultures. A) Cell growth/viability of Rat1 fibroblasts with different MYC status exposed to different concentrations of ellipticine for 48 hours. B) Cell proliferation/viability of HO15.19 cells treated for 96 hours with indicated MYCMI-7 concentrations. A and B) Cell growth/viability was measured using the WST-1 assay. C) SK-N-DZ neuroblastoma cells with *MYCN*-amplification grown in 3D cultures after treatment with 5 μM MYCMI-7 for 2 weeks. Colony formation in agarose was detected by MTT staining and quantified. D) Human Burkitt's lymphoma cells with MYC translocations were treated with 6.25 μM MYCMI-7 for 24 hrs followed by WST-1 assay. p-values DMSO vs. MYCMI-7 for Daudi, Raji, and Mutu; 0.0063, 0.016 and 0.014, respectively. E) Focus formation assay. Primary REFs were transfected with MYC + activated RAS and treated with 0.5 μM MYCMI-7 and grown in agarose for 2 weeks, after which the number of transformed foci were scored. F) Colony formation in agarose of c-MYC +Ras transformed REFs after treatment with 1 μM MYCMI-7 for 2 weeks. p-values DMSO vs. MYCMI-7; 0.00011. A-F) All experiments were performed in triplicates and repeated at least three times. The average values are shown. Statistical analysis was performed using student t-test.*= $p<0.05$, **=$p<0.01$, ***=$p<0.001$, ns=not significant.

Supplemental Figure 4

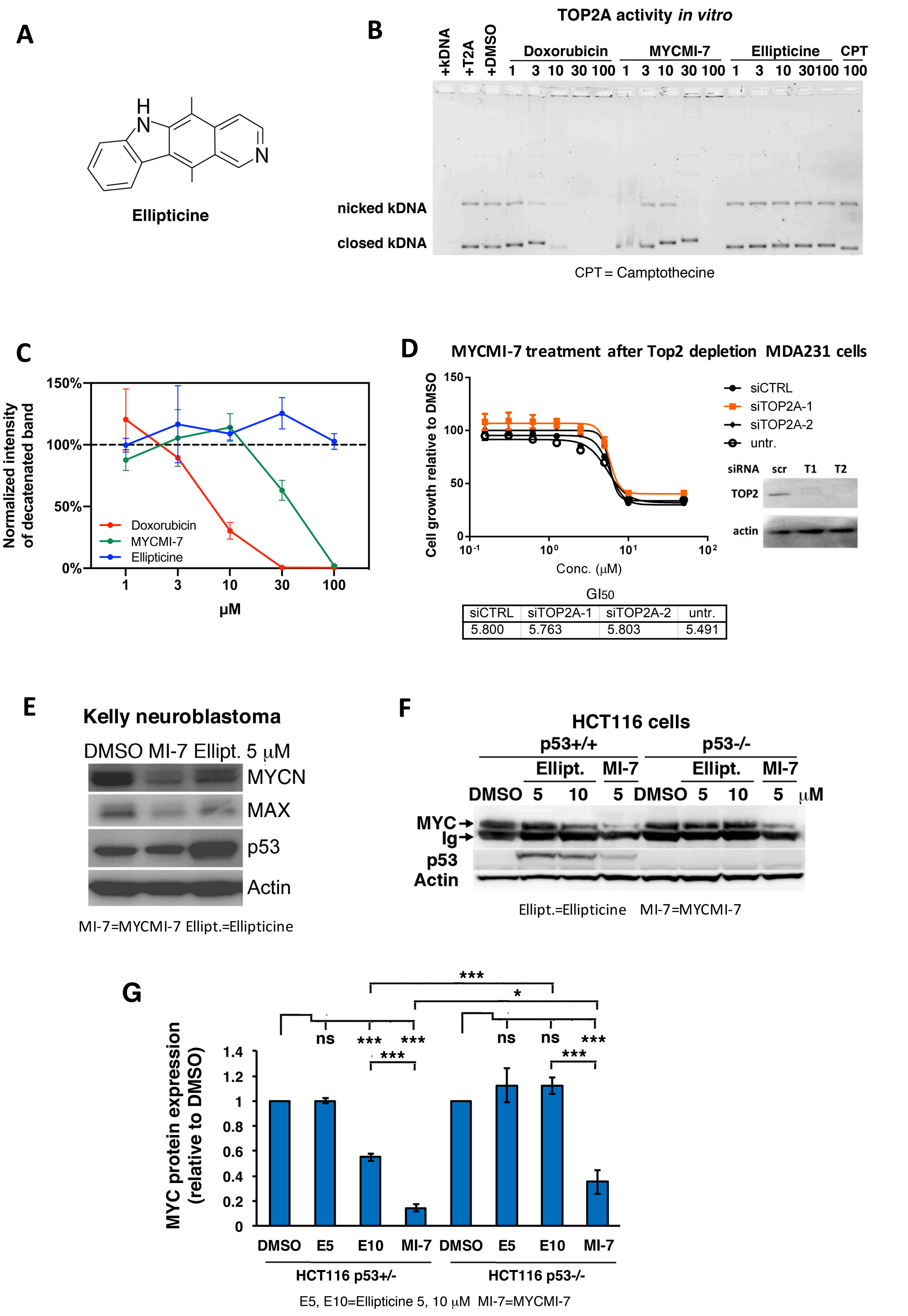


| siCTRL | siTOP2A-1 | siTOP2A-2 | untr. |
|---|---|---|---|
| 5.800 | 5.763 | 5.803 | 5.491 |

**Supplementary Figure 4.** Effects of MYCMI-7 is relation to TOP2A activity *in vitro* and in cells. A) Structure of ellipticine B-C) TOP2A activity and DNA interaction and *in vitro* B) Representative agarose gel of TOP2A activity assay. TOP2 decatenates kinetoplast DNA from interlocking rings unable to penetrate the gel into individual rings that run to the bottom of the gel. The assay was run in the presence of ethidium bromide. C) Plot showing the average of three independent TOP2A activity assays. The decatenated bands detected in A) were quantified and normalized to the decatenated signal from TOP2A + DMSO sample (Lane 3). Error bars represent the standard deviation. D) siRNA-mediated depletion of TOP2A in MDA-MB-231 cells does not affect sensitivity to MYCMI-7 as determined by resazurin assay after titration of MYCMI-7 concentrations is the presence and absence of TOP2A. The western blot confirms downregulation of Top2A in response to siRNA transfection. $GI_{50}$ values are displayed for MYCMI-7 treatment after the different siRNA transfections. E) Western blot analysis of MYCN, MAX and p53 expression after exposure to 5 μM MYCMI-7 or ellipticine overnight in MYCN-amplified Kelly neuroblastoma cells. F) Western blot analysis of p53 expression after exposure to MYCMI-7, and ellipticine overnight at indicated concentrations in p53 wt and p53-/- HCT116 cells. MYC was immunoprecipitated in F) to ensure detection of low levels of MYC after MYCMI-7 treatment. G) Quantification of MYC expression shown in F) normalized to actin expression. p-values DMSO vs. 5 μM ellipticin (E5), 10 μM ellipticin (E10), MYCMI-7 (MI-7) in p53 wt cells; 0.80, 2.20x10-5, 1.17x10-6, respectively, by t-test. p-values DMSO vs. E5, E10 and MYCMI-7 in p53-/- cells; 0.22, 0.051 and 0.00049, respectively. p-value E10 in p53 wt vs. p53-/- cells; 0.00027. p-value MYCMI-7 in p53 wt vs. p53-/- cells; 0.031. p-values E10 vs. MYCMI-7 in p53 wt and p53-/- cells; 0.00011 and 0.00053, respectively.

*= $p<0.05$, **=$p<0.01$, ***=$p<0.001$, ns=not significant.

Suppl. Figure 5

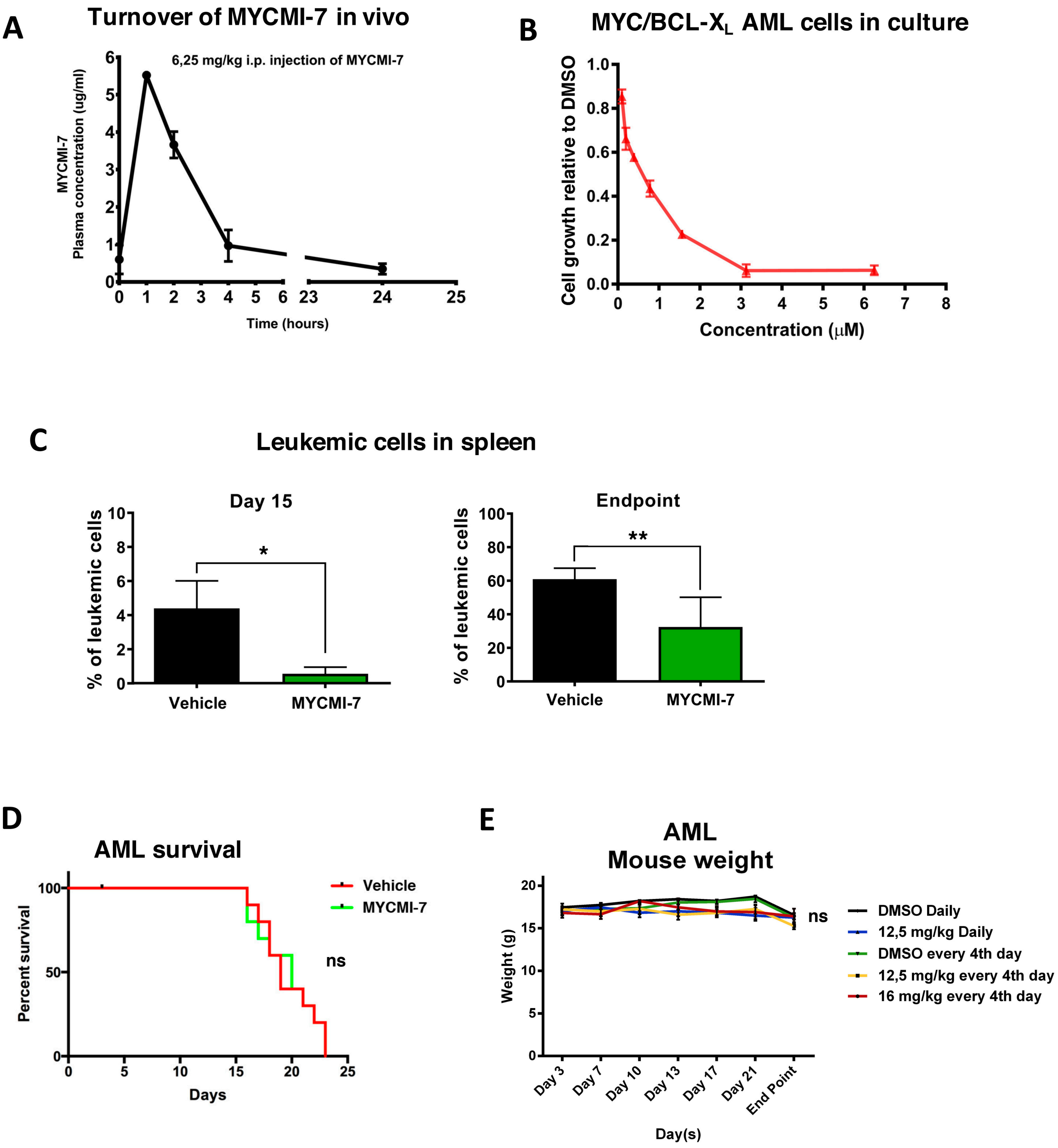

**Supplementary Figure 5.** Treatment of MYC/BCL-XL-driven acute myeloid leukemia with MYCMI-7. A) Measurement of MYCMI-7 turnover in the blood after i.p. injection of 6.25 mg/kg as determined by mass spectrometry. B) Treatment of MYC/BCL-$X_L$ leukemic cells purified from spleens with indicated concentrations of MYCMI-7 in cell culture for 72 hrs. C) Flow cytometry analysis of leukemic blasts in the spleen on day 15 (vehicle; n=5, MYCMI-7; n=3) and at the end point after treatment with 12.5 mg/kg MYCMI-7 (n=5), compared with vehicle (n=5). p-values vehicle vs. MYCMI-7 day 15 and at end point; 0.0165 and 0.0098, respectively, by t-test. D) Survival curve of the mice treated with daily i.p. injections of 12.5 mg/kg MYCMI-7 (n=10) or vehicle (n=10). p-value vehicle vs. MYCMI-7; 0.9698, by log rank test. E) Mouse weights at different time points during experiments testing different protocols for scheduling MYCMI-7 treatment as indicated (n=5 per condition).

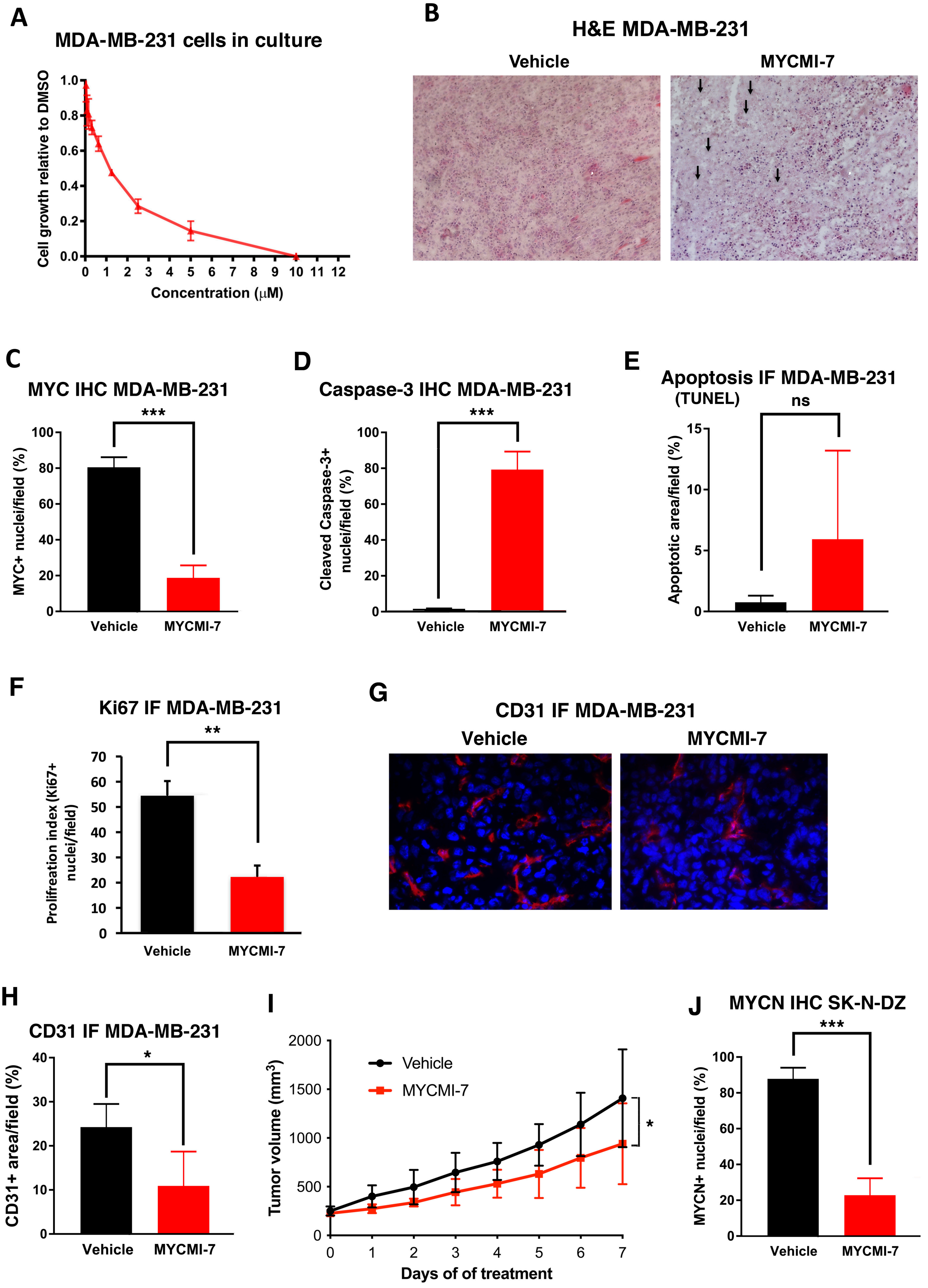
A
MDA-MB-231 cells in culture
Cell growth relative to DMSO
Concentration (μM)
B
H&E MDA-MB-231
Vehicle
MYCMI-7
C
MYC IHC MDA-MB-231
MYC+ nuclei/field (%)
***
Vehicle
MYCMI-7
D
Caspase-3 IHC MDA-MB-231
Cleaved Caspase-3+ nuclei/field (%)
***
Vehicle
MYCMI-7
E
Apoptosis IF MDA-MB-231 (TUNEL)
ns
Apoptotic area/field (%)
Vehicle
MYCMI-7
F
Ki67 IF MDA-MB-231
Prolifreation index (Ki67+ nuclei/field)
**
Vehicle
MYCMI-7
G
CD31 IF MDA-MB-231
Vehicle
MYCMI-7
H
CD31 IF MDA-MB-231
CD31+ area/field (%)
*
Vehicle
MYCMI-7
I
Tumor volume (mm3)
Vehicle
MYCMI-7
*
Days of of treatment
J
MYCN IHC SK-N-DZ
MYCN+ nuclei/field (%)
***
Vehicle
MYCMI-7

**Supplementary Figure 6.** MYCMI-7 inhibits growth of MDA-MB-231 breast cancer cell and SK-N-DZ MYCN-amplified neuroblastoma cell xenografts *in vivo*. A) Treatment of MDA-MB-231 cells with indicated concentrations of MYCMI-7 for in cell culture for 72 hrs. B) H&E stainings of MDA-MB-231 xenograft tumor tissue from vehicle- and MYCMI-7 treated animals at end point. Necrotic/apoptotic areas are indicated with arrows. C and D) Quantification of C) MYC and D) Caspase 3 IHC staining of MDA-MB-231 xenografts from Fig. 8C. p-values vehicle vs. MYCMI-7 for MYC and Caspase 3; 0.0003 and 0.0002, respectively. E and F) Quantification of E) TUNEL and F) Ki67 IF staining of MDA-MB-231 xenografts from Fig. 8D. p-values vehicle vs. MYCMI-7 for Ki67 and TUNEL; 0.003 and 0.1123, respectively. G) CD31 immunofluorescence stainings of MDA-MB-231 xenograft tumor tissue from vehicle- and MYCMI-7 treated animals at end point. H) Quantification of CD31 IF staining of MDA-MB-231 xenografts from G). p-value vehicle vs. MYCMI-7 for CD31; 0.0418. I) MYCN-amplified SK-N-DZ neuroblastoma xenograft mice injected with 6.25 mg/kg MYCMI-7 or vehicle as described in the legend to Fig. 8. Tumor growth during the first 7 days when all mice were still alive. p-value vehicle vs. MYCMI-7 at day 7; 0.0477. J) Quantification of MYCN IHC staining of SK-N-DZ xenografts from Fig. 8H. p-value vehicle vs. MYCMI-7 for MYCN; 0.0006. The statistical analysis in C-J) were performed by t-test. *= p<0.05, **=p<0.01, ***=p<0.001, ns=not significant.
